# Content and design of respectful maternity care training packages for health workers in sub‐Saharan Africa: Scoping review

**DOI:** 10.1002/ijgo.15938

**Published:** 2024-10-30

**Authors:** Judith Yargawa, Marina Daniele, Kelly Pickerill, Marianne Vidler, Angela Koech, Hawanatu Jah, Grace Mwashigadi, Mukaindo Mwaniki, Peter von Dadelszen, Marleen Temmerman, Veronique Filippi, Hannah Blencowe, and the PRECISE‐DYAD Network

**Affiliations:** ^1^ Wolfson Institute of Population Health Queen Mary University of London London UK; ^2^ Maternal, Adolescent, Reproductive & Child Health (MARCH) Centre London School of Hygiene & Tropical Medicine London UK; ^3^ Centre for Maternal and Child Health Research City University of London London UK; ^4^ Department of Obstetrics & Gynaecology University of British Columbia Vancouver British Columbia Canada; ^5^ Department of Obstetrics and Gynecology Aga Khan University Nairobi Kenya; ^6^ Center of Excellence in Women and Child Health Aga Khan University Nairobi Kenya; ^7^ Medical Research Council Unit Fajara The Gambia; ^8^ Department of Women and Children's Health King's College London London UK; ^9^ Department of Obstetrics and Gynaecology and BC Children's Hospital Research Institute University of British Columbia Vancouver British Columbia Canada; ^10^ Faculty of Epidemiology and Population Health London School of Hygiene & Tropical Medicine London UK

**Keywords:** disrespect and abuse, health workers, respectful maternity care, sub‐Saharan Africa, training

## Abstract

**Background:**

Training health workers might facilitate respectful maternity care (RMC); however, the content and design of RMC training remain unclear.

**Objective:**

To explore the content and design of RMC training packages for health workers in sub‐Saharan Africa.

**Search Strategy:**

MEDLINE, EMBASE, CINAHL Complete, Web of Science Core Collections, SCOPUS, and grey literature sources (including websites of RMC‐focused key organizations and Ministries of Health) were searched for journal papers, reports, and training guides from January 2006 up to August 2022.

**Selection Criteria:**

There were no restrictions on study designs, language, or health‐worker cadre. Two reviewers independently screened results.

**Data Collection and Analysis:**

Key data, including training content and methods used, were extracted and summarized.

**Main Results:**

Thirty‐two citations from 26 studies/programs were identified (24 journal papers, 5 manuals/guides, 2 reports and 1 PhD thesis), with 27 citations from 22 studies informing the review findings. About half of all conducted studies were from East Africa. The most common topics in RMC trainings were communication, privacy and confidentiality, and human resources. Most trainings were multicomponent and appear to be largely in‐service training. Health workers providing direct care to women, compared with non‐clinical staff such as receptionists and cleaners, were the only recipients of training in most studies (81.8%). Two broad categories of training methods/tools were identified: workshop‐based and action‐based. Over 90% of the studies assessed impact of the training, with a majority focused on impacts on maternal health and care; however, half of the latter studies did not appear to have feedback mechanisms in place for implementing change.

**Conclusions:**

The content and design of RMC training in sub‐Saharan Africa are multifaceted, suggesting the complexity of implementing/promoting RMC. Some progress has been made; however, missed opportunities in training remain with respect to study populations, training topics, cadres, and feedback mechanisms.

## Synopsis

A scoping review exploring the content, design, methods, trainers, funders, frequency, duration, and evaluation of respectful maternity care training for health workers in sub‐Saharan Africa.

## 1 INTRODUCTION

The improved coverage in institutional births globally is leading to a greater focus on the quality and experiences of care for women and newborns. As a result, respectful maternity care (RMC) has gained significant attention in the past decade. All birth environments should be free from disrespect and abuse (D&A), and actively promote respectful care. To achieve high quality of care, health care should be safe, effective, patient‐centered/acceptable, efficient, accessible, and equitable.^1^ The White Ribbon Alliance has developed an RMC Charter outlining 10 rights to which women and newborns are entitled when receiving maternity care.^2^ The WHO Quality of Care Framework considers provision of care to be just as important as experience of care.^3^

Significant progress has been made in the evidence base on RMC. The Bowser and Hill^4^ landscape study provided a pioneering, comprehensive review of the evidence on D&A, highlighting the issue on a global scale and presenting seven domains of D&A: physical abuse, non‐confidential care, non‐consented care, non‐dignified care, abandonment of care, discrimination, and detention in facilities. Since then, further reviews have been conducted including: typology of mistreatment of women during delivery in health facilities^5^; drivers of mistreatment^6^; impacts of D&A on health outcomes and postnatal care use^7^; women's perspectives on what matters to them during childbirth^8^; experiences of care after stillbirths^9^; perspectives of midwives^10^; tools/instruments for measurement including indicators for routine monitoring and evaluation,^11^ quality assessment,^12^ and general critique of methods used in prevalence studies^13^; prevalence of D&A^14^; effectiveness/impacts of RMC policies and interventions^15^, ^16^, ^17^; and country‐specific reviews and studies.^18^, ^19^, ^20^

Although the knowledge base regarding the prevalence and determinants of RMC/D&A has grown substantially, evidence of intervention and implementation of RMC to address D&A is limited. Health workers lead efforts to provide quality care to women in sometimes difficult working environments^21^, ^22^, ^23^; however, D&A is also at times perpetrated by them. Training health workers and consistently monitoring and evaluating practices can improve RMC.^15^ However, the content and design of these trainings remain unclear. This scoping review aimed to explore the content and design of RMC training packages for health workers in sub‐Saharan Africa. Specific objectives included:

1. To identify the content of RMC training packages for health workers
2. To identify the design of the RMC training packages (including funding)
3. To determine whether or not the training varies by cadre of health workforce
4. To find out whether or not the training is tailored to promote RMC for service users with specific characteristics
5. To investigate whether or not the impact of the training is assessed, the types of evaluations conducted, and the existence of feedback mechanisms for implementing change.

## 2 MATERIALS AND METHODS

### 2.1 Eligibility criteria

Working definitions for key themes in the research topic guided the inclusion and exclusion criteria. Health workers were defined as any cadre providing either direct medical care (for example, doctors, nurses, midwives, community health extension workers) or those involved in wider healthcare operations (for instance, receptionists, porters, cleaners). We considered health workers working in any type of health facility in sub‐Saharan Africa, including primary, secondary, and tertiary health facilities operated by governmental or non‐governmental bodies. Training packages could either promote RMC or aim to reduce D&A.

Inclusion criteria included: studies focused on health‐worker training either promoting RMC or addressing D&A (including studies focusing on only one aspect of RMC, for instance, informed consent); studies on quality‐of‐care training with an RMC component; qualitative and quantitative studies and non‐research sources; studies conducted in sub‐Saharan Africa; published in any language. Exclusion criteria included: quality of care training without an RMC component; training for health workers assisting home deliveries; and editorials or commentaries.

### 2.2 Information sources and search strategy

MEDLINE, EMBASE, CINAHL Complete, Web of Science Core Collections, and SCOPUS were searched from January 1, 2006, up to November 2021. Grey literature sources were searched between November 2021 and August 2022: WHO African Index Medicus; websites of key organizations focused on RMC (White Ribbon Alliance, HEARD Project, Quality of Care Network, International Confederation of Midwives and FIGO [International Federation of Gynecology & Obstetrics]); and websites of Ministries of Health of all 49 sub‐Saharan African countries, including additional sites hosting country data (Table S1). The website searches included tab‐by‐tab, key word, and resource repository searches. Journal papers, reports, training guides/manuals, and other relevant documents were retrieved, and additional sources were also retrieved from reviewing reference lists of included studies from databases.

Synonyms of “respectful maternity care” and “disrespect and abuse” were searched in combination with synonyms of “training” and “health workers” using both Medical Subject Headings (MESH) and free texts (Table S2).

### 2.3 Study selection

Retrieved papers were exported to a central database and managed using Endnote X8 and Covidence, and Microsoft applications for grey literature. Papers were first screened by title, then abstract and full text, with a yes, no, or maybe outcome assigned. Two reviewers screened all papers from the central databases and discrepancies were resolved through discussion. High inter‐rater reliability was observed, with discrepancies in around 2% of screened papers, which were subsequently resolved. As RMC training can be multicomponent and could potentially be linked to numerous topics, an additional set of second‐order inclusion/exclusion criteria were used to guide the screening process (Table S3). A breakdown of studies from central databases excluded at the full‐text screening stage is listed in Table S4.

### 2.4 Data charting, synthesis, and analysis

A data‐charting excel sheet was developed to extract information (Table S5). The sheet was refined through piloting. The scoping review was primarily informed by the typology of RMC developed by Shakibazadeh et al.,^24^ a comprehensive framework that not only covers RMC‐relevant actions by health workers but also incorporates health‐system‐wide, woman‐ and family‐level factors. This typology provides 12 domains of RMC: being free from harm and mistreatment; maintaining privacy and confidentiality; preserving women's dignity; prospective provision of information and seeking informed consent; ensuring continuous access to family and community support; enhancing quality of physical environment and resources; providing equitable maternity care; engaging with effective communication; respecting women's choices that strengthen their capabilities to give birth; availability of competent and motivated human resources; provision of efficient and effective care; and continuity of care.^24^ First, the content of the identified RMC training packages was mapped to these 12 RMC domains, with provision for a 13th “other” category. Two D&A and mistreatment typologies—Bowser and Hill^4^ and Bohren et al.^5^—are widely used in the field and were mapped to the Shakibazadeh framework to further guide data extraction (Table S6).

Data were synthesized descriptively and narratively. In a few studies, the specific content of the training was not explicitly provided but was implicitly inferable from other parts of the paper. Categories and sub‐categories were developed and counts were made within each. Commonalities and heterogeneity across studies, countries, health facility types, health‐worker cadres, and other factors were explored.

### 2.5 Reporting

The review was registered on the Open Science Framework in August 2021.^25^ Findings have been reported in accordance with the PRISMA Extension for Scoping Reviews (PRISMA‐ScR) checklist^26^ (Table S7).

## 3 RESULTS

### 3.1 Study selection

**FIGURE 1:**
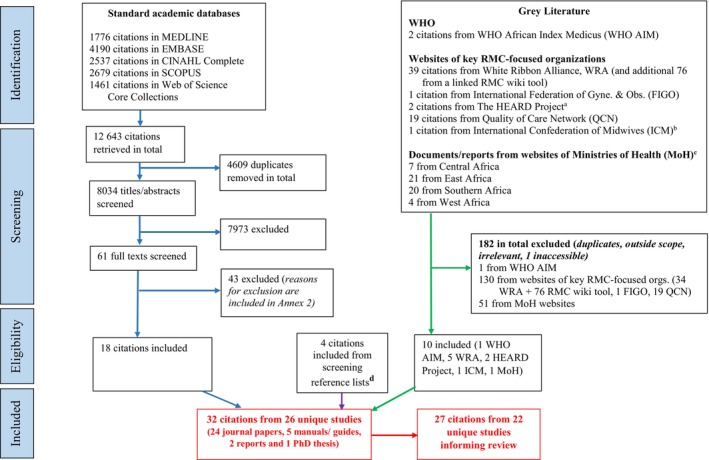
Flow chart of search results from all data sources. a: One citation from the HEARD Project is a pack of 7 manuals/guides and powerpoint presentations from the Heshima Project. These have been counted as one citation in the flowchart, as they are multiple resources within a single study. b: This citation from the ICM is a pack of 3 manuals/guides and powerpoint presentation from the RESPECT toolkit. These have been counted as one citation in the flowchart, as they are multiple resources within a single study. c: Breakdown of MoH reports gotten: East Africa (3 Ethiopia, 6 Kenya, 1 Madagascar, 4 Tanzania, 7 Uganda); Central Africa (3 Equatorial Guinea, 3 Congo Republic, 1 Burundi); Southern Africa (7 Mozambique, 13 South Africa); West Africa (2 Burkina Faso, 1 multi‐country including Guinea, Ghana and Nigeria, and 1 pan‐African). d: 2 training manuals, 1 PhD thesis and 1 journal paper retrieved from screening reference lists.

The database searches yielded 12 643 citations, 61 full texts were screened and 18 studies were included, with four citations included from screening reference lists. A further 10 citations were obtained from the grey literature sources. Across all data sources, 32 citations from 26 studies/programs were included in the review (24 journal papers, 5 manuals/guides, 2 reports and 1 PhD thesis) (Figure 1).

### 3.2 Study characteristics

Of the 32 citations, 27 citations were studies already conducted (labeled as “conducted studies”; Table 1a) and five were manuals/guides (Table 1b). The conducted studies are the focus of this review's findings, with a summary of the manuals/guides included for signposting purposes. Five of the conducted studies were additional citations from studies already incorporated in the review; these provide additional information to the main studies (labeled as “extra studies”; Table 1a). Hence the review includes 22 unique studies using a range of study designs, with before‐and‐after/pre‐post design common. All studies were single‐country studies, and about half were from East Africa (Kenya, Tanzania, Ethiopia, and Rwanda) (Table 1a). Most studies were in public‐sector facilities across a range of levels, although hospitals were predominant.

**TABLE 1a:** Overview of included studies: Conducted studies (*n* = 27 citations, 22 studies).

	General study information						Study area information		
S/N	Study and reference #	Stated aim of study	Country	Study design	Year(s) study conducted	Part of wider training or project yes/no	Setting	Number of HF and level	Type of HF
1.	Abuya et al. 2015^27^	To measure the effect of interventions to reduce the prevalence of D & A during labour and delivery in 13 Kenyan health facilities	Kenya	Before‐and‐after quantitative study	June 2011–Feb 2014	No	Mixed (4 rural and the rest urban or peri‐urban)	13 facilities (including 3 public referral hospitals, 3 district public hospitals with maternity units, 2 faith‐based hospitals, 2 private nursing homes, 1 public health centre)	Mixed (public, private, faith‐based)
	Warren et al. 2017^28^ (Extra study)	To describe and analyse the implementation process of Heshima Project, its strengths and challenges and lessons gained		Qualitative	2011–2016	Yes	Mixed (urban and rural)	13 health facilities (included health centres and hospitals in 5 counties)	
2.	Afulani et al. 2019^29^	To evaluate the effect of an integrated simulation‐based training on RMC provision	Ghana	Pre‐post cross‐sectional study (pilot study)	2017	No	Rural	5 district delivery facilities (1 referral hospital and 4 health centres)	Public (with 1 mission referral hospital)
3.	Akin‐Otiko and Bhengu 2013^30^	To explore an interpersonal communication and counselling (IPCC) capacity building approach to empower midwives for friendly service and result‐oriented client education at first level of midwifery practice	Nigeria	Mixed (both quantitative and qualitative)	2010	No	Mixed (rural, urban and urban slums)	Not reported (but 9 health facilities were selected from 8 of the 23 LGAs in Kaduna, Nigeria)	Not reported
4.	Asefa et al. 2020a^31^	To examine service providers' reaction to and experiences of RMC training and implementation	Ethiopia	Interventional mixed‐methods (pre‐post survey and post‐intervention FGDs)	2018	Yes	Not reported (but in SNNPR)	3 hospitals (1 primary, 2 general hospitals; all comprehensive emergency obstetric care hospitals)	Public
	Asefa et al. 2020b^32^ (Extra study)	To assess women's experiences of mistreatment during facility‐based childbirth before and after implementation of a respectful maternity care intervention		Pre‐post study	Dec 2017–Sept 2018				
5.	Brown et al. 2007^33^	To increase number of women with a companion during childbirth (secondary objective: to improve practice)	South Africa	Cluster randomised trial	1998–1999	No	Urban	10 health facilities (Midwife obstetric units, district hospitals (level 1 hospitals), and referral hospitals (level 2 hospitals)	Public
6.	Dzomeku et al. 2021^34^	To evaluate impact of a 4‐day RMC training in midwives' daily maternity care practices	Ghana	Qualitative	2019	Yes (part of a PhD thesis)	Urban	1 tertiary hospital	Public
	Dzomeku 2016^35^ (Extra study)	To develop an in‐service training program for midwives to provide patient‐centered childbirth care that would increase client satisfaction with childbirth care							
7.	Geddes et al. 2017^36^	To design and pilot an RMC‐promoting training module for clinical midwives (Other aims: To also show link between human rights and maternal health care, and how a human rights‐based approach may improve experiences of patients and care providers)	Malawi	Qualitative (pilot study)	2015	No	Not reported (but follow‐on study title says "rural Malawi")	Not reported	Not reported
8.	Honikman et al. 2020^37^	To engender an ethos of care and compassion within maternity settings, in order to prepare these environments for mental health task‐shifting initiatives	South Africa	Theatre‐inspired	Unclear	No	Not reported	Not reported	Not reported
9.	Mengistu et al. 2021^38^	To describe the development, implementation and results of a range of interventions to improve RMC	Ethiopia	Qualitative	2016–2019	Yes	Rural	17 health centres and 3 primary hospitals (in 3 districts in 3 regions ‐ Tigray, Oromia, and SNNPR)	Public
10.	Mihret et al. 2020^39^	To reduce D&A of mothers during antenatal care and delivery services	Ethiopia	Mixed (pre‐post interventional study and qualitative study)	Nov. 2018–May 2019	No	Unclear (but at Injibara General Hospital)	1 general hospital	Public
11.	Ndayambaje et al. 2017^40^	To estimate effect of the human resources for health midwifery in‐service mentorship model on episiotomy rates	Rwanda	Mixed (pre‐post intervention study and cross‐sectional study)	2012, 2014	Yes	Urban	1 secondary district hospital (the largest maternity specialty hospital in Rwanda)	Public
12.	Okonofua et al. 2020^41^	To improve self‐reported indicators of maternal healthcare satisfaction by women	Nigeria	Quasi‐experimental	2017–2019	Yes	Urban	4 secondary hospitals (2 referral hospitals as intervention, and 2 hospitals as controls; all 4 were either central hospitals or general hospitals)	Public
13.	Oosthuizen et al. 2020^42^	To find out the effect of the 'CLEVER Maternity Care' package, a multi‐faceted intervention to improve respectful, quality obstetric care	South Africa	Before‐and‐after study	2016–2017	Yes	Unclear (but in Tshwane health district)	10 primary facilities (all of them midwife‐led obstetric units, MOUs)	Public
	Oosthuizen et al. 2019^43^ (Extra study)	To implement a multicomponent intervention to change the complex interplay between preventable maternal and perinatal mortality and morbidity and poor clinical governance and supervision in midwife‐led labour units		Mixed methods (quantitative and qualitative)	Jan 2015–Dec 2017				
14.	Ouedraogo et al. 2014^44^	To develop the interpersonal skill of health workers in Burkina Faso. Also to reinforce RMC skills among Society of Gynaecologists and Obstetricians of Burkina Faso members & health workers	Burkina Faso	Qualitative (direct observations)	2012–2013	No	Mixed (rural and urban)	3 health facilities (1 primary rural district hospital, 1 secodary urban regional hospital, and 1 tertiary urban university hospital)	Public
15.	Pfeiffer et al. 2019^45^	To describe the design, implementation and evaluation of an 18 month‐long leadership training and coaching program for health workers	Ghana	Mixed (qualitative and survey)	Jan 2014–June 2015	Yes	Urban	1 tertiary referral hospital	Public
16.	Kujawski et al. 2017^46^	To assess a participatory community and health system intervention to reduce the prevalence of disrespect and abuse during childbirth in Tanzania	Tanzania	Before‐and‐after study	2011–2016	Yes	Rural	2 district hospitals	Public
	Ramsey et al. 2016^47^ (Extra study)	To test approaches to measure prevalence of disrespect and abuse during childbirth, and develop and monitor approaches to reducing it		Mixed (Survey, FGDs and IDIs, observation, project documentation and monitoring)				2 district hospitals (but the wider study included 8 facilities in the 2 districts)	
17.	Ratcliffe et al. 2016^48^	To describe the implementation process and outcomes of two interventions to reduce disrespect and abuse in the study facility	Tanzania	Mixed (pre‐post studies, interviews and direct observations)	Jan 2013–Dec 2014	Yes	Urban	1 tertiary (a regional referral hospital)	Public
18.	Shimoda and Lida 2018^49^	Unclear	Tanzania	Seminar	2018	No	Urban	1 tertiary hospital	Public
19.	Umbeli et al. 2014^50^	To assess impact of health care providers' training on patient‐provider's communication during childbirth in the labour ward	Sudan	Quasi‐interventional study	2011	No	Urban	1 tertiary hospital	Public
20.	Webber et al. 2018^51^	To improve attitudes of health workers towards pregnant women	Tanzania	Qualitative study (a pilot study)	Not reported	No	Unclear (but in Rorya District, Mara Region)	Not reported (but consisted of hospitals, health centres, dispensaries in the district)	Not reported
21.	Wilson‐Mitchell et al. 2018^52^	To develop and deliver a 2‐day RMC workshop for midwives using Intellectual Partnership Model principles	Tanzania	Before‐and‐after study	July–August 2017	No	Rural	Not reported	Not reported
22.	Zethof et al. 2020^53^	To assess recollection of informed consent before and after introducing a multicomponent intervention	Malawi	Pre‐post study	January–June 2018	No	Rural	1 hospital (level not reported)	Mission

**TABLE 1b:** Overview of included studies: Manuals/guides (*n* = 5 citations, 4 studies).

Organization/author	Publication year	Title	Stated purpose	Any target	Guidance given on	Materials/documents included^a^
USAID and MCHIP (Maternal and Child Health Integrated Program)	2015	Respectful Maternity Care Workshop: Learning resource package	To provide guidance and materials for conducting a one‐day RMC workshop for clinicians	Clinicians and clinical supervisors (but also clinical managers and other stakeholders in clinical settings)	Guidance given on: RMC subject, objectives of the workshop, participants, supplies and equipment needed for training session; plan for session including agenda items, role of facilitators and suggested allocated time	Yes
White Ribbon Alliance	2015	Respectful Maternity Care: A Nigeria‐focused health workers' training guide	“To support communities and, specifically, healthcare providers in confronting D&A during facility‐based childbirth and promoting dignity in evidence‐based maternity care.” Adapted from a generic Population Council guide (Ndwiga et al., 2015), it was designed “to reflect the Nigerian context and the specific needs of healthcare workers at primary, state, and federal levels in the country”	Everyone. Health facility managers and providers at all levels of the system Designed to be a useful tool for a wide range of stakeholders in pre‐service, in‐service and advocacy	This domesticated guide was specially tailored to the Nigerian context. Designed for standalone RMC workshops or for incorporation into regular activities (e.g. monthly facility seminars)	Yes
International Confederation of Midwives	2020	RESPECT workshops: A toolkit RESPECT workshops: Facilitator's guide RESPECT workshops: PowerPoint slides	“To help raise awareness about how crucial RMC is and to encourage others to think critically about their own and others behaviour amongst those providing maternity services.” This is a 3‐in‐1 package consisting of the RESPECT toolkit, the RESPECT workshop PowerPoint slides and the RESPECT facilitator's guide	Maternity care workers (whether as individuals or as a group), but added can be used by anyone e.g. midwives, doulas, doctors, researchers, policy‐makers, managers, advocates, etc	Background, using the toolkit, activities, RMC policies/guides, Reading list, useful web‐links and videos, sample lesson plans Being a facilitator and tips, workshop preparations, practical steps, expressing the RESPECT workshop vision, and self‐reflection The PowerPoint slides are already‐prepared and include detailed, helpful notes in the notes pane	Yes
Maternal and Child Survival Program, USAID (Currie, S.)	2016	Alternative birth positions	To provide materials for sensitization, training and follow up on alternative birth positions as a key component of implementing RMC	Maternity workers/providers	Session outline, background/overview (including rationale for supporting births in alternative positives, skills demonstration), useful references/ resources, role‐play guidance, pictures and instructions for supporting birth on ‘all‐fours’, and a job aid with pictures showing many different labour and birth positions	Yes
Population Council (Ndwiga et al.)	2014‐ 2016	Larger publication: Respectful maternity care resource package (*from Heshima Project)* Consists of: Promoting respectful maternity care: A training guide for community‐based workshops (Community facilitator's guide) Promoting respectful maternity care: A training guide for facility‐based workshops (Facilitator's guide) Promoting respectful maternity care: A training guide for facility‐based workshops (Participant's guide) Promoting respectful maternity care resource package: Community flipchart Heshima lessons learned brief PowerPoint presentations (Promoting respectful maternity care (rmc) at birth: Orientation for community‐based workshops; Promoting respectful maternity care (rmc) at birth: Orientation for facility‐based workshops)	“To promote increased support, advocacy, and provision of high‐quality, woman‐centred maternity care” Tailor‐designed to be conducted at the facility level and also at the community level	A wide range: Supervisors, program managers, clinicians, service providers, community health workers, technical advisers, policy makers, trainers, communities legal and health rights advocates, media professionals, civil right groups, society leaders, etc	A wide range of topics including overview of maternal health, rights, the RMC subject, roles in promoting RMC for different stakeholders, monitoring and data management, etc. Also includes references/ links to other resources, action plans, role‐play scripts, workshop schedule, forms, worksheets, exit interview questionnaire for clients, pamphlets/brochures, etc	Yes

^a^ For example, forms, worksheets/exercises, role‐play scripts, questionnaires, PowerPoint slides, training schedule, etc.

### 3.3 Objective 1: To identify the content of RMC training packages for health workers

Most trainings were multicomponent and somewhat complex, and included a broad range of aims including reducing D&A, changing practice, and improving maternal satisfaction (Table 2). The most frequent RMC topics were effective communication (seven studies, domain #8), maintaining privacy and confidentiality (six studies, domain #2), availability of competent and motivated human resources (six studies, domain #10), prospective provision of information and seeking informed consent (five studies, domain #4), and ensuring continuous access to family and community support (five studies, domain #5). Table S8.1 shows specific examples of training content for each RMC domain.

**TABLE 2:** Content of RMC training packages for health workers by RMC domains (*n* = 27 citations, 22 studies).

S/N	Study	RMC Domain #1	RMC Domain #2	RMC Domain #3	RMC Domain #4	RMC Domain #5	RMC Domain #6	RMC Domain #7	RMC Domain #8	RMC Domain #9	RMC Domain #10	RMC Domain #11	RMC Domain #12
1.	Abuya et al. 2015^27^; Warren et al. 2017^28^												
2.	Afulani et al. 2019^29^												
3.	Akin‐Otiko and Bhengu 2013^30^												
4.	Asefa et al., 2020^31^, ^32^												
5.	Brown et al. 2007^33^												
6.	Dzomeku et al. 2021 & 2016^34^, ^35^												
7.	Geddes et al. 2017^36^												
8.	Honikman et al. 2020^37^												
9.	Mengistu et al. 2021^38^												
10.	Mihret et al. 2020^39^												
11.	Ndayambaje et al. 2017^40^												
12.	Okonofua et al. 2020^41^												
13.	Oosthuizen et al. 2020 & 2019^42^, ^43^												
14.	Ouedraogo et al. 2014^44^												
15.	Pfeiffer et al. 2019^45^												
16.	Kujawski et al. 2017^46^; Ramsey et al. 2016^47^												
17.	Ratcliffe et al. 2016^48^												
18.	Shimoda and Lida 2018^49^												
19.	Umbeli et al. 2014^50^												
20.	Webber et al. 2018^51^												
21.	Wilson‐Mitchell et al. 2018^52^												
22.	Zethof et al. 2020^53^												

*Note*: Green represents “included”; Red represents “not included.” The RMC Domains (Shakibazadeh et al., 2018): #1: Being free from harm and mistreatment; #2: Maintaining privacy and confidentiality; #3: Preserving women's dignity; #4: Prospective provision of information and seeking informed consent; #5: Ensuring continuous access to family and community support; #6: Enhancing quality of physical environment and resources; #7: Providing equitable maternity care; #8: Engaging with effective communication; #9: Respecting women's choices that strengthens their capabilities to give birth; #10: Availability of competent and motivated human resources; #11: Provision of efficient and effective care; #12: Continuity of care. Studies not mapped to any domain were included in the 13th ‘other’ category.

Additional RMC‐related topics not easily fitting into our analytical framework were included in some trainings^24^ (Table S8.2). These included topics on rights (e.g. human rights, patients'/providers' rights), codes of conduct/ethics, attitudes (e.g. empathy, interpersonal skills, relationship development with women), and non‐specific RMC topics (e.g. compassionate care, patient‐centered care, prevalence of mistreatment in the setting). Beyond RMC, other topics sometimes related broadly to maternal health care (e.g. focused antenatal care, basic emergency obstetric and newborn care (BEmONC), maternal death reviews and surveillance, birth preparedness), and healthcare management (e.g. leadership, inter‐professional collaboration, problem‐solving).

### 3.4 Objective 2: To identify the design of the RMC training packages

Similar to content of the training (Objective 1), the methods/tools used in the training were multicomponent.

Two broad categories of methods/tools were identified: workshop‐based and action‐based (Table 3). The workshop‐based category was common and consisted of presentations, modules, and didactic sessions. A wide range of participatory approaches were used in these sessions to facilitate learning, for example, role plays, interactive discussions, case studies, group work, ice‐breakers, and story‐telling. Written materials were sometimes provided and a range of tools were used to aid learning. The action‐based category included a broad range of activities that went beyond information transfer to practical implementation (“learning‐by‐doing” initiatives) and activities targeted at particular groups (health workers, the community, and policy makers). A range of actions were carried out to promote RMC and tackle D&A in health facilities including establishment of quality improvement teams, providing suggestion boxes in facilities, and putting up wall posters in maternity wards. There were actions focused on health workers including pastoral support activities (e.g. counseling, mentorship, and coaching), guidance/monitoring activities (supervisory and follow‐up visits), and incentives/benefits. Other activities were focused on women or the community such as community sensitization workshops, Maternity Open Days (to build trust with the community and help dispel myths/misconceptions about facility delivery), monitoring/resolving D&A cases and counseling for victims of D&A. Additional initiatives were aimed at policy makers/facility leaders and included stakeholder forums, consultative meetings for RMC buy‐in, and continuous dialogue.

**TABLE 3:** Methods/tools used in RMC training across studies (*n* = 27 citations, 22 studies).

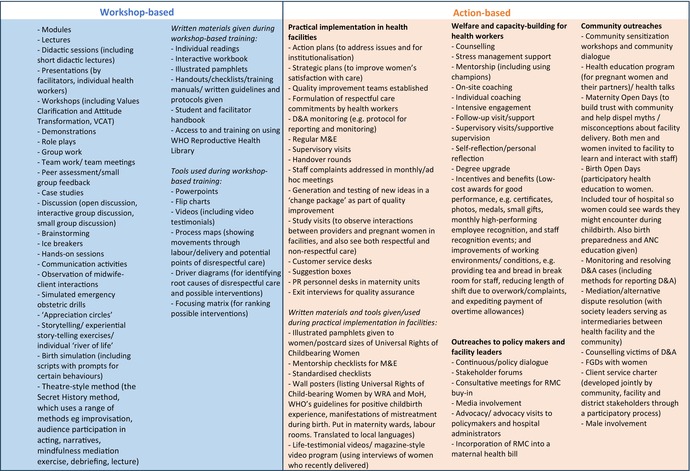

The frequency and duration of training varied across studies (Table 4). Workshops were more commonly conducted as one‐off activities whereas process‐type activities (e.g. supervisory visits, mentorship and counseling for providers) tended to be done multiple times at specified or continuous intervals. Duration of training varied from a few hours or days (e.g. workshops and meetings) to several months (e.g. quality improvement processes).

**TABLE 4:** Frequency and duration of RMC training package activities (*n* = 27 citations, 22 studies).

Type of training activity	Frequency	Duration^a^
Workshops	Mainly one‐off Workshop was the most common one‐off training activity Refresher workshops were done multiple times (including monthly)	Most workshops lasted between 2‐3 days, with 3 days more popular. Workshops lasting for ≤1 day (e.g. for 3 hours), within 4‐6 days or carried out throughout a specified duration (2 months) were also reported. For refresher trainings, this lasted for a few hours.
Meetings	Multiple	These mainly related to quality improvement activities, which were often organized routinely/continuously. Done weekly in some studies and quarterly in others. Also specified as lasting for <1 day.
Community‐related events	One‐off: Community dialogues and stakeholder meetings Multiple: Maternity open days	Maternity Open Days, community dialogue meeting, stakeholder forum lasted for 1 day
Monitoring & evaluation, supervisory and follow‐up visits	Multiple Supervisory visit was the most common training activity done multiple times. Intervals for supervisory visits included monthly. Every 2 weeks: Monitoring & evaluation	A range of durations including <1 day, 4‐6 days, and within 2‐5 months.
Counselling and mentorship for providers	Multiple	The duration of counselling was not often apparent but one study^28^ mentioned that it lasted for 45min‐ 1 hour per session. Mentorship tended to be done routinely/continuously or over a longer period of time (>1 year)
Quality improvement processes	Multiple	This lasted over a prolonged duration: 6‐ 12 months.
Others	—	Duration for preparatory training work was also provided in some studies, for example, development of RMC resources, curricula, client service charter or the intervention. This included a range of durations including 1 week, 1 month and 6‐12 months.

^a^ As durations were reported in diverse ways in the papers, the categories within this column were created to facilitate reporting.

Other aspects of design of the RMC training packages that were considered included: commissioners, funders, and facilitators of the training; modes and location of the training; attendance option; and incentives for attendance. We found that some details about the training were not reported in most studies. This included whether attendance was mandatory or optional, mode (face‐to‐face, online, or hybrid, although the trainings appear to have been largely face‐to‐face), and incentives for attendance. Data regarding the commissioner and location of the training were missing or unclear in around half of studies.

Commissioners and funders of RMC training in sub‐Saharan Africa are diverse and commonly involve multiple organizations/institutions including international organizations, governments, and universities/research institutes. International organizations were listed as funders in 72.7% of the studies (Table S8.3). Training organizations and personnel were mainly researchers/academics, health providers, international organizations, government/policy makers, and local non‐governmental organizations/groups and they cumulatively played a variety of roles including developing/reviewing training materials and/or implementing or facilitating the training (Table S8.3). For studies reporting location of training, this was mostly on‐site, with one study^45^ justifying this as cost‐effective, leading to maximum clinical staff participation. The trainings were largely in‐service training only, except in two studies in which midwifery students were included.^40^, ^49^ Regarding incentives for training, this appeared to be largely part of the action‐based training. Excluding these, one study^49^ mentioned that certificates were given to participants during a Certificate Ceremony at the end of the seminar, and another study^31^ mentioned that participants were given compensation for transportation.

### 3.5 Objective 3: To determine whether or not the training varies by cadre of health workforce

Data were not available on specific training methods by health worker cadre. However, the results suggest that trainings were delivered to a variety of cadres (Table S8.4). Health workers providing direct care to women, compared with those involved in hospital operations/administration, were the only recipients of training in most studies (81.8%). The health‐worker cadre most commonly included in training was midwives (68.2%), followed by doctors/physicians/surgeons (45.5%), then nurses and other/broad clinical groupings (both 36.4%); non‐clinical roles made up only 13.6% of the training cadre. More than half (63.6%) of trainings in the included studies involved mixed cadres in the training group.

### 3.6 Objective 4: To establish whether the training is tailored to promote RMC for specific characteristics of service users

Almost all studies designed the training to promote RMC for all women, rather than tailoring it to specific characteristics of service users such as educational level/socioeconomic status, age, residence, disability, ethnicity, and birth outcome. There were a few exceptions. A few studies incorporated specific characteristics of women (e.g. “difficult” patient, a young adolescent mother, or a non‐cooperative woman during labor) in role‐play and simulation activities conducted during training. Two studies focused on specific patient characteristics to align with the studies' training aims: women undergoing cesarean section only (aimed at improving the obtaining of informed consent before cesarean section),^53^ and women who had vaginal births only (aimed at reducing episiotomy rates).^40^

### 3.7 Objective 5: To investigate whether or not the impact of the training is assessed, evaluations are conducted, and feedback mechanisms for implementing change exist

Over 90% of the studies assessed impact of the training including impacts on maternal health and care (72.7% of studies) and impacts on health‐worker‐related metrics such as knowledge and experience (36.4% of studies) (Table 5). In all, 18.2% evaluated both categories.

**TABLE 5:** Overview of training evaluations conducted in included studies (*n* = 27 citations, 22 studies).

Category	Frequency (%)
**Impact of training evaluated on any category**	
Yes	20 (90.9)
No	2 (9.1)
**Impact of training evaluated on maternal health and care**	
Yes	16 (72.7)
No	5 (22.7)
Somewhat/unclear	1 (4.5)
**Impact of training evaluated on health worker‐related metrics (knowledge, experience, etc )**	
Yes	8 (36.4)
No	13 (59.1)
Somewhat/unclear	1 (4.5)
**Both impact of training on maternal health/care and health worker‐related metrics evaluated**	
Yes	4 (18.2)
No	16 (72.7)
Somewhat/unclear	2 (9.1)
**Methods used in evaluation (for impact on maternal health and care; *n* = 16)**	
Quantitative	8 (50.0)
Qualitative	1 (6.3)
Mixed	6 (37.5)
Unclear	1 (6.3)
**Methods used in evaluation (for impact on health worker‐related metrics; *n* = 8)**	
Quantitative	0 (0.0)
Qualitative	2 (25.0)
Mixed	6 (75.0)
Unclear	0 (0.0)
**Feedback mechanisms in place (for impacts on maternal health and care; *n* = 16)**	
Yes	7 (43.8)
No	8 (50.0)
Somewhat/unclear	1 (6.3)

Half of the studies evaluating impacts on maternal health/care used quantitative methods, 6.3% qualitative, and a little over one‐third (37.5%) used mixed methods. Of the studies using quantitative methods, more than half (62.5%) used a before‐and‐after/quasi‐experimental design, with several conducting exit surveys/exit interviews with women. The studies using qualitative methods used a number of designs including observations, focus group discussions, in‐depth interviews, case narratives, and document analysis. The mixed methods studies used a combination of these two methods, and sometimes included process evaluations. Mixed methods were used predominantly for the studies assessing impact on health‐worker‐related metrics (75%). Common quantitative designs included pre‐post studies/tests and usage of assessment form/evaluation form/questionnaire/survey to collate feedback from the health workers, and common qualitative designs included focus group discussions and in‐depth interviews.

Half of the studies evaluating impacts on maternal health/care did not appear to have feedback mechanisms in place for implementing change. Studies without feedback mechanisms tended to be those using before‐and‐after/pre‐post designs involving exit surveys with women, and those with feedback mechanisms tended to be using process evaluation measurements. A wide variety of feedback mechanisms were noted in studies including written mentorship feedback given to units, supervisory/follow‐up visits, discussion with staff following observations of midwife‐client interactions, discussions in routine meetings (with inclusion of a standing item on respectful care in morning meetings), inclusion of maternity staff in quality improvement teams, meetings, complaints mechanisms, and regular monitoring of progress towards goals. It was less clear whether studies evaluating impacts on health‐worker‐related metrics had feedback mechanisms in place for implementing change. Specific evaluation results on impacts of the training are beyond the scope of this review.

## 4 DISCUSSION

This comprehensive scoping review identified 22 unique studies of RMC educational interventions or training for health workers in 10 sub‐Saharan African countries, plus 5 manuals/guides for national or international use. Many training packages were part of multicomponent interventions and the topics covered related to most of the domains for promotion of RMC and prevention of D&A of Shakibazadeh et al.'s framework.^24^ A range of group workshops and individual training or mentoring sessions were offered. Action plans and a variety of initiatives were implemented at the facility and community levels. In over three‐quarters of the trainings, health workers providing direct care to women were the only recipients of the training, which excluded operations/administrative staff. Nearly two‐thirds of the trainings were directed at mixed cadres, with midwives the most frequently included group. With two exceptions, all trainings focused on all maternity service users rather than particular sub‐groups. Over 90% of studies conducted some form of impact evaluation. Three‐quarters focused on health and care impacts, about one‐third on health‐worker‐related impacts and one‐fifth on both. Half the studies evaluating impact on health and care did not have feedback mechanisms in place.

We surveyed 16 years of published outputs with a comprehensive search strategy, aiming to capture all relevant publications from before the conceptualization of RMC/D&A by Bowser and Hill.^4^ We considered training content beyond specific acts of respectful/disrespectful care and also included multicomponent training designs. In spite of the multicomponent and heterogeneous nature of the RMC trainings, we attempted to synthesize and categorize the data in meaningful ways. Our findings are specific to sub‐Saharan Africa, which improves the relevance to African countries; however, RMC trainings in other parts of the world may be different. Our review was unable to capture trainings that had not been written up, either as academic papers or as reports that could be identified via the grey literature search. As we have identified primarily academic papers and reports, the trainings included here may be among the most carefully conceptualized, as demonstrated by the high proportion that were evaluated.

There is overlap between our findings and those of other reviews of multicomponent RMC policies and interventions,^15^, ^17^ because most of the latter include a training component. In addition, Dhakal et al.^54^ published a systematic review of educational interventions to promote RMC in 2022, covering both high‐ and low‐income countries. Our review and these other reviews are complementary—the others, by focusing on rigorous evaluations, have largely examined effectiveness, whereas ours has aimed to provide a comprehensive overview of the design and characteristics of training programs, with a specific focus on sub‐Saharan Africa. Our review included double the number of unique studies compared with Dhakal et al.,^54^ perhaps because our search included additional data sources. Our findings can raise awareness of existing initiatives and their specific features among stakeholders at international, national, and sub‐national levels, potentially avoiding duplication of efforts and supporting the future intervention design.

The number of training packages identified suggests that the RMC agenda has risen in prominence in recent years in the sub‐Saharan African region; however, the fact that only 10 countries in sub‐Saharan Africa (approximately 20% of the total) were represented, demonstrates a dearth of evidence. It is plausible that health workers are being trained on RMC in other countries, but these trainings have not been captured for research purposes. In terms of content, the inclusion of broad topics related to RMC such as rights, ethics, and professional attitudes is a welcome development. As mistreatment in maternity care is a symptom of systemic disempowerment and abuse against women and children,^55^ the explicit inclusion of gender‐based inequities and violence could be a useful addition to future programs. Further, it is well established that service users who are socioeconomically vulnerable, adolescents, or migrants are at higher risk of experiencing disrespect and abuse.^56^, ^57^ For this reason, it is disappointing that the topic of equitable care did not appear to be addressed in any training programs, although it is plausible that this was covered under broad training topics such as ethics, rights, and attitudes in Domain #13 (Table S8.2). Combined with the limited evidence of provision of RMC training focused on specific service user groups, this suggests that most training initiatives to date lack a well‐developed social justice and equity perspective. Although not explicitly mentioned in Shakibazadeh et al.'s framework,^24^ respectful newborn care and respectful stillbirth care were also not apparently covered.^58^ While acknowledging that some training content may not have been documented in publications, we urge that these important topics be integrated into future training programs.

The lack of published evidence on pre‐service training is disappointing. Post‐registration training is key to creating clinical practice environments that embody RMC in which both students and newly qualified staff can learn,^59^, ^60^ but RMC training needs to commence at the onset and be embedded within curricula in medical, nursing, and midwifery institutions.

A commendable feature of most trainings was that multiprofessional teams were trained together, given that mutual respect and collaboration based on a shared vision are essential for the provision of quality care.^61^ However, operations and administrative staff, also part of the maternity system with which women come in contact (e.g. receptionists), were not included; future training should include these in all facilities where such cadres exist. Another issue is lack of clarity around who the trainers were and the extent to which a service user perspective was included in the trainings, although the latter may have been included in baseline studies. The lived experience of service users can be immensely powerful in changing hearts and minds and reducing the perceived “otherness” of the population served. This perspective can be presented to participants both in reported and pre‐recorded form, or, preferably, in the form of a live service user trainer or speaker.^62^

In terms of evaluation, exit interviews were commonly used, and carry the risk of recall bias, social desirability bias, and courtesy bias. In addition, other authors have highlighted a lack of high‐quality or standardized measures available to assess respectful and disrespectful maternity care in low‐ and middle‐income countries.^12^, ^13^ In view of these considerations, the inclusion of mixed methods of data collection in many studies was a positive feature and is to be recommended for future interventions, as no method alone can be considered as standard.^63^

Previous reviews have provided evidence of the effectiveness of well‐designed educational initiatives, mostly as part of multicomponent interventions, to improve RMC.^15^, ^17^, ^54^ However, the relative effectiveness of different training approaches has not been established, such as individual sessions versus group workshops, as well as what might be the benefits and drawbacks of combining training with different intervention components. Nevertheless, the search for a single best approach that suits all contexts may be misguided, as even carefully crafted global tools require adaptation and contextualization to maximize relevance and appropriateness. Overall, participatory training interventions involving active engagement, collaboration, and reflective practice are more likely to be effective, compared with lecture‐style presentations.^64^ Longer‐term, embedded programs with follow up and/or regular updates, as implied by the term “continuous”, are also likely to have greater impact.^65^

In practice, the success of any form of health‐worker training may depend on a range of considerations related to both the facility and the health system, such as the presence of effective local champions, buy‐in at multiple levels within the organization (from frontline staff to hospital leadership), staff attendance and turnover, cost considerations, and the existence of external support and other mechanisms to overcome local organizational difficulties.^66^ Furthermore, effective RMC training may be challenging to achieve as the structural drivers of D&A are deep‐rooted.^67^ The working conditions and remuneration for health workers in many settings are below acceptable standards. These include midwives operating at the bottom of institutional hierarchies, being underpaid, practicing in unsafe environments, and lacking equipment and opportunities for self‐development.^10^ Midwives in Ethiopia, for example, were critical of one RMC training program in that it helped to center women's rights, but did not take their own into consideration.^31^ Until some of these structural barriers are addressed, training—whether or not in conjunction with other components—is unlikely to eradicate the problem.

Finally, while training programs remain financed through international aid, sustainability remains problematic.^68^ Ideally, health systems in sub‐Saharan Africa should be able to plan and financially sustain the training required by their staff. In addition, the multiplication of donor‐driven staff trainings has exacerbated human resource shortages and created inequitable training cultures focused around per‐diem compensation.^69^ In‐service RMC training should be embedded in a system approach that takes a holistic and rational view of the staff's training needs, while centering the service‐user experience.^70^ The development of feedback mechanisms that ensure all stakeholders, including frontline workers and the local population, can benefit from the results of evaluations is not only morally imperative, but will also facilitate a sense of ownership of the process, increasing the likelihood of sustainability.

In conclusion, the content and design of RMC training in sub‐Saharan Africa are multifaceted, suggesting the complexity of implementing/promoting RMC. Some progress has been made; however, missed opportunities in training remain in terms of study populations, training topics, cadres, and feedback mechanisms. Training programs are distributed unequally, with nearly half of studies conducted in East Africa; more work is needed in other parts of the continent. The inclusion of key information on content and design of training, as well as shareable materials in RMC training studies, could enable shared learning and potential replication of training in other settings. It is imperative that studies assess the impact of training and that these findings are used to inform policy and practice, to drive change towards promoting RMC for every woman everywhere.

## AUTHOR CONTRIBUTIONS

The review was conceptualized by HB, JY, and VF. MV, AK, HJ, MD, KP, GM, JY, HB, MT, and VF provided feedback on the design and protocol of the review. JY, KP, and MD conducted the searches; HB screened reference lists of database sources. MD and JY carried out double‐screening and resolution of discrepancies. JY conducted data charting and data analysis, with feedback from HB. VF, MM, MD, MV, and HB provided feedback on preliminary results. PvD conceptualized and is the principal investigator of the funded projects that supported this work. The manuscript was drafted by JY and MD. All authors reviewed and approved the final manuscript.

## FUNDING INFORMATION

This review is part of the PRECISE‐DYAD work funded by the NIHR–Wellcome Partnership for Global Health Research Collaborative Award, reference 217123/Z/19/Z.

## CONFLICT OF INTEREST STATEMENT

The authors have no conflicts of interest.

## REGISTRATION

This scoping review was registered on the Open Science Framework in August 2021 (https://osf.io/v6hn2).

## Supporting information


Table S1–S8.4.

## Data Availability

Data sharing is not applicable to this article as no new data were created or analyzed in this study.
