# Supplementary material for: Content and design of respectful maternity care training packages for health workers in sub‐Saharan Africa: Scoping review
Source: Int J Gynaecol Obstet. 2024 Oct 30;168(3):857–74. doi: 10.1002/ijgo.15938 (PMC11823297; doi:10.1002/ijgo.15938)
Supplement: Supplementary file 1 — Table S1–S8.4. [file IJGO-168-857-s001.docx]

Appendices

Table of Contents

[**Table S1**: Sub-Saharan African country^*^ list, Ministry of Health websites and additional links searched 2](#_Toc163247288)

[**Table S2**: MEDLINE search strategy (Jan 2006- 8^th^ Nov 2021) 6](#_Toc163247289)

[**Table S3**: 2^nd^ order inclusion/exclusion criteria 9](#_Toc163247290)

[**Table S4**: Reasons for exclusion at full-text stage 10](#_Toc163247291)

[**Table S5^*^**: Data charting excel form 18](#_Toc163247292)

[**Table S5.1**: Data charting excel form for journal papers and reports 18](#_Toc163247293)

[**Table S5.2**: Data charting excel form for training manuals 19](#_Toc163247294)

[**Table S6^*^**: RMC and D&A typology overlaps between Shakibazadeh et al. (2018), Bowser and Hill (2010) and Bohren et al. (2015) 20](#_Toc163247295)

[**Table S7**: Preferred Reporting Items for Systematic reviews and Meta-Analyses extension for Scoping Reviews (PRISMA-ScR) checklist 21](#_Toc163247296)

[**Table S8**: Additional results 23](#_Toc163247297)

[**Table S8.1**: Examples of training content for health workers by RMC domains (source: Shakibazadeh et al. 2018) (n= 27 citations, 22 studies) 23](#_Toc163247298)

[**Table S8.2**: Breakdown of training content outside scope of the 12 RMC domain framework 26](#_Toc163247299)

[**Table S8.3**: Overview of training stakeholders in included studies (n= 27 citations, 22 studies) 27](#_Toc163247300)

[**Table S8.4**: Overview of trainees in included studies (n= 27 citations, 22 studies) 29](#_Toc163247301)

### **Table S1**: Sub-Saharan African country^*^ list, Ministry of Health websites and additional links searched

| **Region** | **Country** | **Ministry of Health website(s)** |
| --- | --- | --- |
| **CENTRAL AFRICA**  **(n=9)** | Burundi | <http://minisante.bi/> |
|  | Cameroun | <https://www.minsante.cm/>  <https://www.minsante.cm/site/?q=en>  <https://www.minsante.cm/site/?q=fr/cat%C3%A9gories/d%C3%A9veloppement-des-ressources-humaines> |
|  | Central Africa Republic | <https://www.msp-centrafrique.net/> |
|  | Chad | <http://www.sante-tchad.org/> |
|  | Congo Republic | <https://sante.gouv.cg/> |
|  | Democratic Republic of Congo | <https://minisanterdc.cd/>  <https://sante.gouv.cd/> |
|  | Equatorial Guinea | https://guineasalud.org/ |
|  | Gabon | <http://www.sante.gouv.ga/> |
|  | Sao Tome and Principe | <http://saude.portal-stp.net/spip.php?rubrique1> |
| **EAST AFRICA**  **(n=14)** | Comoros | <https://beit-salam.km/>  (No dedicated MOH website found but above is a govt. website) |
|  | Djibouti | <https://sante.gouv.dj/publications> |
|  | Eritrea | <https://shabait.com/>  (No dedicated MOH website found but above is a govt. website) |
|  | Ethiopia | <https://www.moh.gov.et/site/> |
|  | Kenya | <https://www.health.go.ke/resources> |
|  | Madagascar | <http://www.sante.gov.mg/ministere-sante-publique/> |
|  | Mauritius | <https://health.govmu.org/Pages/default.aspx> |
|  | Rwanda | <https://www.moh.gov.rw/>  (*A few documents not downloadable)* |
|  | Seychelles | <http://www.health.gov.sc/> |
|  | Somalia | <https://moh.gov.so/> |
|  | South Sudan | <https://moh.gov.ss/> |
|  | Sudan | <http://www.fmoh.gov.sd/En/> |
|  | Tanzania | <https://www.moh.go.tz/en/>  <https://hmisportal.moh.go.tz/hmisportal/#/home>  <http://hidl.afya.go.tz/#/library/dashboard/published-list> |
|  | Uganda | <https://www.health.go.ug/> |
| **Region** | **Country** | **Ministry of Health website(s)** |
| **WEST AFRICA**  **(n=16)** | Benin Republic | https://sante.gouv.bj/ |
|  | Burkina Faso | https://www.sante.gov.bf/accueil |
|  | Cape Verde | https://www.minsaude.gov.cv/ |
|  | Cote d’Ivoire | https://www.sante.gouv.ci/ |
|  | Gambia | <https://www.moh.gov.gm/> |
|  | Ghana | https://www.moh.gov.gh/ |
|  | Guinea | <http://sante.gov.gn/> |
|  | Guinea-Bissau | <https://www.gov.gw/> |
|  | Liberia | https://moh.gov.lr/ |
|  | Mali | http://www.sante.gov.ml/ |
|  | Mauritania | <https://www.sante.gov.mr/> |
|  | Niger | <https://www.sante.gouvne.org/> |
|  | Nigeria | https://www.health.gov.ng/ |
|  | Senegal | https://www.sante.gouv.sn/ |
|  | Sierra Leone | https://mohs.gov.sl/ |
|  | Togo | https://sante.gouv.tg/ |
| **SOUTHERN AFRICA**  **(n=10)** | Angola | <https://minsa.gov.ao/ao/>  <https://minsa.gov.ao/ao/documentos/publicacoes/>  <http://www.inis.ao/> |
|  | Botswana | <https://www.moh.gov.bw/> |
|  | Eswatini | <https://www.gov.sz/index.php/ministries-departments/ministry-of-health> |
|  | Lesotho | <http://health.gov.ls/> |
|  | Malawi | <http://www.health.gov.mw/> |
|  | Mozambique | <https://www.misau.gov.mz/>  <http://mocambique.eportuguese.org/> |
|  | Namibia | <https://mhss.gov.na/> |
|  | South Africa | <https://www.health.gov.za/> |
|  | Zambia | <https://www.moh.gov.zm/> |
|  | Zimbabwe | <http://www.mohcc.gov.zw/> |
| **Additional links searched** | | |
| http://ghdx.healthdata.org/organizations/ministry-public-health-burundi  http://ghdx.healthdata.org/organizations/ministry-public-health-and-fight-against-aids-burundi  https://www.devex.com/organizations/ministere-de-la-sante-publique-et-de-la-lutte-contre-le-sida-ministry-of-public-health-and-the-fight-against-aids-burundi-120712  http://ghdx.healthdata.org/organizations/ministry-public-health-cameroon  https://www.devex.com/organizations/ministry-of-public-health-cameroon-52487  http://ghdx.healthdata.org/organizations/ministry-public-health-and-population-central-african-republic  http://ghdx.healthdata.org/organizations/ministry-public-health-chad  https://www.devex.com/organizations/ministry-of-public-health-chad-52510  https://socialprotection.org/connect/stakeholders/chad-minist%C3%A8re-de-la-sant%C3%A9-publique-ministry-health  http://ghdx.healthdata.org/organizations/ministry-health-congo-rep  http://ghdx.healthdata.org/organizations/ministry-public-health-congo-dr  https://www.devex.com/organizations/ministry-of-health-democratic-republic-of-the-congo-125138  http://ghdx.healthdata.org/organizations/ministry-health-and-social-welfare-equatorial-guinea  http://ghdx.healthdata.org/organizations/ministry-health-gabon  http://ghdx.healthdata.org/organizations/ministry-health-and-public-hygiene-gabon  http://ghdx.healthdata.org/organizations/ministry-health-sao-tome-and-principe  http://ghdx.healthdata.org/organizations/ministry-health-social-cohesion-solidarity-and-gender-promotion-comoros  http://ghdx.healthdata.org/organizations/ministry-public-health-and-population-comoros  https://www.afro.who.int/countries/comoros  https://socialprotection.org/connect/stakeholders/comoros-minist%C3%A8re-de-la-sant%C3%A9-de-la-solidarit%C3%A9-de-la-coh%C3%A9sion-sociale-et-de-la  http://ghdx.healthdata.org/organizations/ministry-health-djibouti  https://healthresearchweb.org/en/djibouti/projects  http://ghdx.healthdata.org/organizations/ministry-health-eritrea  https://www.healthresearchweb.org/en/eritrea/institution_61  https://www.who.int/workforcealliance/countries/eri/en/  https://e-library.moh.gov.et/library/wp-content/uploads/2021/07/13_Ethiopian_Hospital_Alliance_for.pdf  http://ghdx.healthdata.org/organizations/ministry-public-health-madagascar  http://ghdx.healthdata.org/organizations/ministry-health-and-family-planning-madagascar  http://ghdx.healthdata.org/organizations/ministry-health-and-wellness-mauritius  https://healthresearchweb.org/en/mauritius/institution_110  <http://ghdx.healthdata.org/organizations/ministry-health-rwanda>  <http://ghdx.healthdata.org/organizations/ministry-health-seychelles>  <http://ghdx.healthdata.org/organizations/puntland-ministry-health-somalia>  <https://data2.unhcr.org/en/partners/view/302>  <http://ghdx.healthdata.org/organizations/ministry-health-south-sudan>  <http://ghdx.healthdata.org/organizations/ministry-health-sudan>  <https://healthresearchweb.org/en/sudan/>  <https://ghdx.healthdata.org/organizations/ministry-health-and-social-welfare-tanzania>  <https://ghdx.healthdata.org/organizations/ministry-health-uganda>  <https://ghdx.healthdata.org/organizations/ministry-health-angola>  <https://ghdx.healthdata.org/organizations/ministry-health-botswana>  <https://ghdx.healthdata.org/organizations/ministry-health-eswatini>  <https://ghdx.healthdata.org/organizations/ministry-health-and-social-welfare-lesotho>  <https://www.up.ac.za/centre-for-maternal-fetal-newborn-and-child-healthcare/article/2811559/other-projects>  <https://ghdx.healthdata.org/organizations/ministry-health-malawi>  <https://ghdx.healthdata.org/organizations/ministry-health-mozambique>  <https://ghdx.healthdata.org/organizations/ministry-health-and-social-services-namibia>  <https://ghdx.healthdata.org/organizations/department-health-south-africa>  <https://ghdx.healthdata.org/organizations/ministry-health-zambia>  <https://ghdx.healthdata.org/organizations/ministry-health-and-child-welfare-zimbabwe> | | |

Source: United Nations Statistics Division

^*^ Mayotte and Reunion have been excluded as they are French territories, hence part of the Euro Zone. Sudan is listed under “Northern Africa and Western Asia” but we have included the country in this list as it is widely considered to be part of Sub-Saharan Africa geographically.

A few sites were not working

### **Table S2**: MEDLINE search strategy (Jan 2006- 8^th^ Nov 2021)

1. exp Patient-Centered Care/

2. exp Professional-Patient Relations/ or exp Physician-Patient Relations/ or exp Nurse-Patient Relations/ or exp "Attitude of Health Personnel"/

3. exp Quality Improvement/ or exp Quality Assurance, Health Care/

4. "Quality of Health Care"/

5. respectful maternity care.mp.

6. (respect* adj3 care).mp.

7. (digni* adj3 care).mp.

8. (wom?n centr* adj3 (care or healthcare)).mp.

9. (person centr* adj3 (care or healthcare)).mp.

10. (patient centr* adj3 (care or healthcare)).mp.

11. (wom?n orient* adj3 (care or healthcare)).mp.

12. (person orient* adj3 (care or healthcare)).mp.

13. (patient orient* adj3 (care or healthcare)).mp.

14. (qualit* adj3 care).mp.

15. (experienc* adj3 care).mp.

16. (qualit* adj3 improv*).mp.

17. (disrespect* adj3 care).mp.

18. (disrespect* adj3 abus*).mp.

19. mistreat*.mp.

20. (obstetric* adj3 violen*).mp.

21. (neglect* adj3 care).mp.

22. (power* adj3 abus*).mp.

23. (physical* adj3 abus*).mp.

24. (verbal* adj3 abus*).mp.

25. (sex* adj3 abus*).mp.

26. (emotion* adj3 abus*).mp.

27. ((non-digni* or nondigni*) adj3 care).mp.

28. ((non-consent* or nonconsent*) adj3 care).mp.

29. ((non-confidential* or nonconfidential*) adj3 care).mp.

30. Discrimination, Psychological/

31. (psycholog* adj3 discrimin*).mp.

32. (detain* or detent*).mp.

33. (abandon* adj3 care).mp.

34. or/1-33

35. exp Education, Medical, Continuing/ or exp Inservice Training/ or exp Training Support/ or exp Staff Development/

36. exp Clinical Competence/ or exp Education, Medical/ or exp "Internship and Residency"/ or exp Education, Nursing/ or exp Students, Nursing/ or exp Education, Medical, Undergraduate/ or exp Students, Medical/ or exp Teaching/

37. exp Teaching/ or exp Teaching Materials/ or exp Hospitals, Teaching/ or exp Remedial Teaching/ or exp Teaching Rounds/

38. exp Internet-Based Intervention/ or exp Psychosocial Intervention/

39. Health Education/ or Curriculum/

40. ((pre-service or preservice) adj3 train*).mp.

41. ((in-service or inservice) adj3 train*).mp.

42. ((teach* or train*) adj3 interven*).mp.

43. workshop*.mp.

44. mentor*.mp.

45. (on-the-job adj train*).mp.

46. or/35-45

47. exp Maternal Health Services/

48. exp Health Personnel/ or exp Personnel, Hospital/ or exp Medical Staff, Hospital/ or exp Nursing Staff, Hospital/

49. exp Physicians/

50. exp Nurses/ or exp Nursing Staff, Hospital/ or exp Nurse Practitioners/ or exp Nurse Administrators/ or exp Nurse Midwives/ or exp Family Nurse Practitioners/ or exp Nurse Clinicians/ or exp Nurse Anesthetists/ or exp Nursing Research/ or exp Clinical Nursing Research/

51. exp Practice Patterns, Physicians'/ or exp Practice Patterns, Nurses'/ or exp Community Health Nursing/

52. exp Midwifery/

53. exp Community Health Workers/

54. exp Allied Health Personnel/

55. exp "Personnel Staffing and Scheduling"/

56. (health adj3 (worker* or personnel* or profession* or provid* or staff* or manag*)).mp.

57. ((health-care or healthcare) adj3 (worker* or personnel* or profession* or provid* or staff* or manag*)).mp.

58. (health facilit* adj3 (worker* or personnel* or profession* or provid* or staff* or manag*)).mp.

59. (hospital* adj3 (worker* or personnel* or profession* or provid* or staff* or manag*)).mp.

60. (medic* adj3 (worker* or personnel* or profession* or provid* or staff* or manag*)).mp.

61. (matern* adj3 (worker* or personnel* or profession* or provid* or staff* or manag*)).mp.

62. (maternity-care adj3 (worker* or personnel* or profession* or provid* or staff* or manag*)).mp.

63. (obstetric* adj3 (worker* or personnel* or profession* or provid* or staff* or manag*)).mp.

64. (obstetric-care adj3 (worker* or personnel* or profession* or provid* or staff* or manag*)).mp.

65. (labo?r deliver* adj3 (worker* or personnel* or profession* or provid* or staff* or manag*)).mp.

66. (labo?r deliver* adj3 nurse*).mp.

67. (birth adj attendant*).mp.

68. (communit* health* adj3 worker*).mp.

69. ("CHEWs" or "CHEW").mp.

70. (health* extension* adj3 worker*).mp.

71. ("HEWs" or "HEW").mp.

72. ((health or healthcare or health-care) adj3 auxiliar*).mp.

73. (allied adj3 (health or healthcare or health-care)).mp.

74. (doctor* or physician*).mp.

75. obstetrician*.mp.

76. nurse*.mp.

77. midwi?e*.mp.

78. or/47-77

79. exp "Africa South of the Sahara"/

80. (sub-saharan adj africa).mp.

81. (subsaharan adj africa).mp.

82. Angola/ or Benin/ or Botswana/ or Burkina Faso/ or Burundi/ or Cameroon/ or Cameroun/ or Cape Verde/ or Cabo Verde/ or Central African Republic/ or Chad/ or Comoros/ or Congo/ or Brazzaville/ or Cote d'Ivoire/ or Ivory Coast/ or Democratic Republic of Congo/ or DRC/ or Zaire/ or Djibouti/ or Equatorial Guinea/ or Eritrea/ or Eswatini/ or Ethiopia/ or Gabon/ or Gambia/ or Ghana/ or Guinea/ or Guinea-Bissau/ or Bissau/ or Kenya/ or Lesotho/ or Liberia/ or Madagascar/ or Malawi/ or Mali/ or Mauritania/ or Mauritius/ or Mozambique/ or Namibia/ or Niger/ or Nigeria/ or Rwanda/ or "Sao Tome and Principe"/ or Senegal/ or Seychelles/ or Sierra Leone/ or Somalia/ or South Africa/ or South Sudan/ or Sudan/ or Swaziland/ or Tanzania/ or Togo/ or Uganda/ or Zambia/ or Zimbabwe/

83. (Angola or Benin or Botswana or Burkina Faso or Burundi or Cameroon or Cameroun or Cape Verde or Cabo Verde or Central African Republic or Chad or Comoros or Congo or Brazzaville or Cote d'Ivoire or Ivory Coast or Democratic Republic of Congo or DRC or Zaire or Djibouti or Equatorial Guinea or Eritrea or Eswatini or Ethiopia or Gabon or Gambia or Ghana or Guinea or Guinea-Bissau or Bissau or Kenya or Lesotho or Liberia or Madagascar or Malawi or Mali or Mauritania or Mauritius or Mozambique or Namibia or Niger or Nigeria or Rwanda or "Sao Tome and Principe" or Senegal or Seychelles or Sierra Leone or Somalia or South Africa or South Sudan or Sudan or Swaziland or Tanzania or Togo or Uganda or Zambia or Zimbabwe).mp.

84. or/79-83

85. 34 and 46 and 78 and 84

86. limit 85 to yr="2006 -Current"

### **Table S3**: 2^nd^ order inclusion/exclusion criteria

**Include**

- Non-maternal health focused health facility-wide training that includes RMC training
- RMC training integrated within wider quality of care training or quality improvement (i.e., QoC training incorporating RMC, or RMC training included within the context of wider QoC training/activities)
- Focused on health workers’ experiences of training but also describing content/design of the training/interventions
- Considered materials on social justice and rights with training (but excluded if just ‘manifestos’ or advocacy materials)
- Trainings that may sound clinical but with clear RMC angle (e.g. trainings/interventions to reduce routine episiotomy)

**Exclude**

- Health facility- and department-wide training as a whole package ‘lumped into one’ where it wasn’t possible to extract maternal health/RMC training. Also QoC training as part of general health facility or departmental training without RMC/MH component
- Predominantly QoC-focused and only mentioning an RMC component in passing with no content to extract
- Focused on health workers’ experiences of training but only discussing the experiences and not describing content/design of the training/interventions
- Trainings focused on improving access to care rather than RMC
- Focused on health workers perceptions of concepts e.g. acceptability of humanised care
- Training focused on hard clinical skills (e.g. clinical ways to manage postpartum haemorrhage)
- ‘Generalist’ training manuals and maternal health guides (for example, guides on managing complications in pregnancy and childbirth, or BMoc, EMoc and CMoc papers that were very clinical)
- Broad, non-maternal health trainings

### **Table S4**: Reasons for exclusion at full-text stage

* All reasons that apply ticked

| **Paper** | **Title** | **Quality of care training without RMC component** | **Other training or activities without RMC component (e.g. on hard clinical skills)** | **Broad, general training (including non-MH contexts)** | **Study already included (i.e. repetition)** | **Non-full text output (e.g. protocol, abstract)** | **Ineligible participants** | **Editorial/ commentary** | **Focused on perceptions of concepts, experiences, non-training topics, etc** | **Any other comment** |
| --- | --- | --- | --- | --- | --- | --- | --- | --- | --- | --- |
| Afulani et al., 2019 | Can integrated clinical simulation trainings improve personcentred maternity care? Results from a pilot project in Ghana |  |  |  | X | X |  |  |  | Abstract. Full study already selected (Afulani et al., 2019) |
| Afulani et al., 2020 | Provider knowledge and perceptions following an integrated simulation training on emergency obstetric and neonatal care and respectful maternity care: A mixed-methods study in Ghana |  |  |  | X |  |  |  |  | Paper focused on experiences and knowledge of staff following the training/intervention in Afulani et al., 2019 (already selected) |
| Amanya 2017 | Community health workers' training in Uganda: The living goods model |  | X |  |  | X |  |  |  | Abstract. Training also appears to be broad, non-RMC specific |
| Archer and Meyer, 2018 | Interventions aimed towards the development of patient-centredness in undergraduate medical curricula: A scoping review |  |  | X |  |  |  |  |  |  |
| Asefa et al., 2020 | Mitigating the mistreatment of childbearing women: evaluation of respectful maternity care intervention in Ethiopian hospitals |  |  |  | X |  |  |  |  | Selected paper: Asefa et al., 2020 |
| **Paper** | **Title** | **Quality of care training without RMC component** | **Other training or activities without RMC component (e.g. on hard clinical skills)** | **Broad, general training (including non-MH contexts)** | **Study already included (i.e. repetition)** | **Non-full text output (e.g. protocol, abstract)** | **Ineligible participants** | **Editorial/ commentary** | **Focused on perceptions of concepts, experiences, non-training topics, etc** | **Any other comment** |
| Baltzell et al., 2017 | Collaborative nursing leadership field course in Malawi |  | X |  |  | X | X |  |  | Training participants also include US nurses |
| Blake et al., 2016 | Training for emergency obstetrical care across the world: The ALARM Programs |  | X |  |  | X | X |  |  | Abstract. Includes training in Canada, but program has an international arm. Training has social justice but on sexual and reproductive rights, with hard clinical skills quite prominent. |
| Brady et al., 2019 | The contribution of midwifery education to the  provision of woman-centred care: Outcomes and implications of an integrative review |  |  |  |  | X | X |  | X | All but one studies included in the review are from high-income settings |
| Delamou et al., 2017 | Building training and research capacities in Ebola affected countries: the case of the Belgian Cooperation in Guinea |  |  | X |  | X |  |  |  | Abstract |
| Dickinson et al., 2019 | Improving the quality of midwifery training in resource limited settings: A quasi-experimental study |  |  |  |  | X |  |  |  | Abstract |
| **Paper** | **Title** | **Quality of care training without RMC component** | **Other training or activities without RMC component (e.g. on hard clinical skills)** | **Broad, general training (including non-MH contexts)** | **Study already included (i.e. repetition)** | **Non-full text output (e.g. protocol, abstract)** | **Ineligible participants** | **Editorial/**  **commentary** | **Focused on perceptions of concepts, experiences, non-training topics, etc** | **Any other comment** |
| Dominico et al., 2018 | Sustainable improvement in healthcare providers' EMONC skills through mentorships in rural Tanzania |  |  |  |  | X |  |  |  | Abstract |
| Goto et al., 2017 | Mid-term evaluation results of improvement in medical interventions and midwifery care in five medical regions in Senegal |  |  |  |  | X |  |  |  | Abstract |
| Habib et al., 2020 | Promoting respectful maternity care for adolescents in Ghana: a quasi-experimental  study protocol |  |  |  |  | X |  |  |  | Protocol |
| Jones et al., 2018 | Rebuilding people-centred maternal health services in post-Ebola Liberia through participatory action research | X |  |  |  |  |  |  |  | Focus seems to be improving access to care rather than RMC |
| Kamiru et al., 2009 | Effectiveness of a training program to increase the  capacity of health care providers to provide HIV/  AIDS care and treatment in Swaziland |  |  | X |  |  | X |  |  | Training is on HIV/AIDs care for pediatric patients |
| Kananura et al., 2017 | Participatory monitoring and evaluation approaches that influence decision-making:  lessons from a maternal and newborn study in Eastern Uganda |  |  | X |  |  |  |  |  |  |
| **Paper** | **Title** | **Quality of care training without RMC component** | **Other training or activities without RMC component (e.g. on hard clinical skills)** | **Broad, general training (including non-MH contexts)** | **Study already included (i.e. repetition)** | **Non-full text output (e.g. protocol, abstract)** | **Ineligible participants** | **Editorial/ commentary** | **Focused on perceptions of concepts, experiences, non-training topics, etc** | **Any other comment** |
| Katowa-Mukwato et al., 2021 | Implementing Evidence Based Practice nursing using the PDSA model: Process, lessons and implications |  |  | X |  |  |  |  |  |  |
| Kermode et al., 2017 | Walking Together: Towards a collaborative model for maternal health care in pastoralist communities of Laikipia and Samburu, Kenya |  | X |  |  |  |  |  |  | Focused on the development of an SBA/TBA collaborative care model, rather than RMC training for HCPs. Model is currently being piloted. |
| Kpangaala-flomo et al., 2021 | Structure, Process, and Outcomes of Liberian National Nursing and Midwifery Curricular Revisions |  | X |  |  |  |  |  |  | Focused on general nursing and midwifery curricula revision |
| Lalonde et al., 2020 | FIGO collaboration for safe and respectful maternity care |  |  |  |  |  |  | X |  |  |
| Larson et al., 2019 | Effect of a maternal and newborn health system quality improvement project on the use of facilities for childbirth: a cluster-randomised study in rural Tanzania | X |  |  |  |  |  |  |  |  |
| Larson et al., 2020 | Did a quality improvement intervention improve quality of maternal health care? Implementation evaluation from a cluster-randomized controlled  study | X |  |  |  |  |  |  |  |  |
| **Paper** | **Title** | **Quality of care training without RMC component** | **Other training or activities without RMC component (e.g. on hard clinical skills)** | **Broad, general training (including non-MH contexts)** | **Study already included (i.e. repetition)** | **Non-full text output (e.g. protocol, abstract)** | **Ineligible participants** | **Editorial/ commentary** | **Focused on perceptions of concepts, experiences, non-training topics, etc** | **Any other comment** |
| Mayers 2007 | Introducing human rights and health into a nursing curriculum |  |  | X |  |  |  |  |  |  |
| McGiven et al., 2017 | ‘Pastoral practices’ for quality improvement in a Kenyan clinical network |  |  | X |  |  | X |  |  | Pediatrics-focused |
| Msibi et al., 2014 | Establishing a national programme for continuing professional development of nurses and midwives in Swaziland |  | X |  |  | X |  |  |  | Brief report. On exploratory work. Focused on broad CPD |
| Mutayoba et al., 2015 | Institutionalization of standard based management and recognition process for quality MNH services |  | X |  |  | X |  |  |  | Abstract (included in Covidence but not found in conference list). Focus appears to be broad, non-RMC related |
| Mwakatundu et al., 2018 | Lessons learned from 10 years of improving access and quality emonc services in rural Tanzania |  |  |  |  | X |  |  |  | Abstract |
| Mukwenda et al., 2017 | Enhancing core competencies and improving midwifery quality of care in lake zone, Tanzania | X |  |  |  | X |  |  |  | Abstract |
| Nayiga et al., 2014 | Strengthening patient-centred  communication in rural  Ugandan health centres:  A theory-driven evaluation  within a cluster randomized trial |  |  | X |  |  |  |  |  | Training not within MH context; was on health workers in public health centres |
| **Paper** | **Title** | **Quality of care training without RMC component** | **Other training or activities without RMC component (e.g. on hard clinical skills)** | **Broad, general training (including non-MH contexts)** | **Study already included (i.e. repetition)** | **Non-full text output (e.g. protocol, abstract)** | **Ineligible participants** | **Editorial/ commentary** | **Focused on perceptions of concepts, experiences, non-training topics, etc** | **Any other comment** |
| Nelson et al., 2012 | Evaluation of a novel training package among frontline maternal, newborn, and childhealth workers in South Sudan |  | X |  |  |  |  |  |  |  |
| Ngabonzima et al., 2020 | Developing and implementing a novel mentorship model (4+ 1) for maternal, newborn and child health in Rwanda |  | X |  |  |  |  |  |  |  |
| Ngoma et al., 2021 | Improving quality of intrapartum care in zambia by expanding on helping babies survive (HBS) and helping mothers survive(HMS) training using project echo (Extension for Community Healthcare Outcomes) |  |  |  |  | X |  |  |  | Abstract. Training described also appears to be broad. |
| Pirkle et al., 2014 | Training and nutritional components  of PMTCT programmes associated with improved intrapartum quality of care in Mali and Senegal |  | X |  |  |  |  |  |  | Focus not on review’s aim. Plus QoC measured using clinical audit, hence no RMC component |
| Plowright et al., 2018 | Formative evaluation of a training intervention for community health workers in  South Africa: A before and after study |  |  | X |  |  |  |  |  |  |
| **Paper** | **Title** | **Quality of care training without RMC component** | **Other training or activities without RMC component (e.g. on hard clinical skills)** | **Broad, general training (including non-MH contexts)** | **Study already included (i.e. repetition)** | **Non-full text output (e.g. protocol, abstract)** | **Ineligible participants** | **Editorial/ commentary** | **Focused on perceptions of concepts, experiences, non-training topics, etc** | **Any other comment** |
| Ratcliffe et al., 2016 | Applying a participatory approach to the promotion of a culture of respect during  childbirth |  |  |  | X |  |  | X |  | Selected paper: Ratcliffe et al., 2016 |
| Reynolds et al., 2017 | Building a maternal and newborn care training programme for health-care professionals in Guinea-Bissau |  | X |  |  |  |  |  |  | Focus of training was on ‘hard’ clinical skills |
| Sibley et al., 2014 | Improving Maternal and Newborn Health Care Deliveryin Rural Amhara and Oromiya Regions of Ethiopia Through the Maternal and Newborn Health in Ethiopia Partnership | X |  |  |  |  |  |  |  |  |
| Sundby and Cham, 2009 | Capacity building in maternal health research in an Africancountry–15 years of experience |  |  |  |  | X |  |  |  | Abstract |
| Vesel et al., 2015 | Psychosocial support and resilience building among health workers in Sierra Leone: interrelations between coping skills, stress levels, and interpersonal relationships |  |  | X |  |  |  |  |  |  |
| Villadsen et al., 2015 | Antenatal care strengthening for improved quality of care in Jimma, Ethiopia: an  effectiveness study | X |  |  |  |  |  |  |  | Predominantly QoC-focused; RMC component mentioned in passing. |
| **Paper** | **Title** | **Quality of care training without RMC component** | **Other training or activities without RMC component (e.g. on hard clinical skills)** | **Broad, general training (including non-MH contexts)** | **Study already included (i.e. repetition)** | **Non-full text output (e.g. protocol, abstract)** | **Ineligible participants** | **Editorial/ commentary** | **Focused on perceptions of concepts, experiences, non-training topics, etc** | **Any other comment** |
| Wilson-Mitchell et al., 2018 | Overview of literature on RMC and applications to Tanzania |  |  |  |  |  |  |  | X | Provided overview on lit, including on non-training topics such as conceptualisation of RMC, and RMC methodologies/ frameworks |

* A few documents in French were retrieved and assessed by bilingual team members; these did not meet the inclusion criteria.

### **Table S5^*^**: Data charting excel form

#### **Table S5.1**: Data charting excel form for journal papers and reports

**This has been split into 2 for space purposes. Comments were also included to guide data charting; these were deleted to improve readability of the sheet.*

#### **Table S5.2**: Data charting excel form for training manuals

### **Table S6^*^**: RMC and D&A typology overlaps between Shakibazadeh et al. (2018), Bowser and Hill (2010) and Bohren et al. (2015)

| **S/N** | **Shakibazadeh et al. (2018)**  ***12 RMC domains*** | **Bowser and Hill (2010)**  ***7 D&A domains*** | **Bohren et al. (2015)**  ***7 mistreatment domains*** |
| --- | --- | --- | --- |
| 1. | Being free from harm and mistreatment | Physical abuse, non-dignified care, detention | Physical abuse, verbal abuse, sexual abuse |
| 2. | Maintaining privacy and confidentiality | Non-confidential care | Failure to meet professional standards of care,  Health system conditions and constraints |
| 3. | Preserving women’s dignity | Non-dignified care | Failure to meet professional standards of care, Poor rapport between women and providers |
| 4. | Prospective provision of information and seeking informed consent | Non-consented care | Failure to meet professional standards of care |
| 5. | Ensuring continuous access to family and community support | ----- | Poor rapport between women and  providers |
| 6. | Enhancing quality of physical environment and resources | ----- | Health system conditions and  constraints |
| 7. | Providing equitable maternity care | Discrimination | Stigma and discrimination |
| 8. | Engaging with effective communication | ----- | Poor rapport between women and  providers |
| 9. | Respecting women’s choices that strengthens their capabilities to give birth | ----- | Poor rapport between women and  providers |
| 10. | Availability of competent and motivated human resources | ----- | Health system conditions and  constraints |
| 11. | Provision of efficient and effective care | Abandonment of care | Failure to meet professional standards of care |
| 12. | Continuity of care | Abandonment of care | Failure to meet professional standards of care |

* A few themes in Bowser and Hill (2010) and Bohren et al. (2015) cut across more than one domain in Shakibazadeh et al. (2018) and were maintained accordingly. In addition, a few themes in Bowser and Hill and Bohren et al. were not mentioned explicitly in Shakibazadeh et al., for example, ‘detention’, ‘physical abuse’, ‘sexual abuse’ and some aspects of ‘health system conditions and constraints’ (i.e., facility culture of bribery and extortion, unclear fee structures, and unreasonable requests of women by health workers). These were placed in the Shakibazadeh domains that most appropriately or comprehensively capture these descriptors.

### **Table S7**: Preferred Reporting Items for Systematic reviews and Meta-Analyses extension for Scoping Reviews (PRISMA-ScR) checklist

| **SECTION** | **ITEM** | **PRISMA-ScR CHECKLIST ITEM** | **REPORTED ON PAGE #** |
| --- | --- | --- | --- |
| **TITLE** | | | |
| Title | 1 | Identify the report as a scoping review. | 1 |
| **ABSTRACT** | | | |
| Structured summary | 2 | Provide a structured summary that includes (as applicable): background, objectives, eligibility criteria, sources of evidence, charting methods, results, and conclusions that relate to the review questions and objectives. | 2-3 |
| **INTRODUCTION** | | | |
| Rationale | 3 | Describe the rationale for the review in the context of what is already known. Explain why the review questions/objectives lend themselves to a scoping review approach. | 5 |
| Objectives | 4 | Provide an explicit statement of the questions and objectives being addressed with reference to their key elements (e.g., population or participants, concepts, and context) or other relevant key elements used to conceptualize the review questions and/or objectives. | 5, 6 |
| **METHODS** | | | |
| Protocol and registration | 5 | Indicate whether a review protocol exists; state if and where it can be accessed (e.g., a Web address); and if available, provide registration information, including the registration number. | 9 |
| Eligibility criteria | 6 | Specify characteristics of the sources of evidence used as eligibility criteria (e.g., years considered, language, and publication status), and provide a rationale. | 6-7 |
| Information sources* | 7 | Describe all information sources in the search (e.g., databases with dates of coverage and contact with authors to identify additional sources), as well as the date the most recent search was executed. | 6-7 |
| Search | 8 | Present the full electronic search strategy for at least 1 database, including any limits used, such that it could be repeated. | 7, Table S2 |
| Selection of sources of evidence† | 9 | State the process for selecting sources of evidence (i.e., screening and eligibility) included in the scoping review. | 7 |
| Data charting process‡ | 10 | Describe the methods of charting data from the included sources of evidence (e.g., calibrated forms or forms that have been tested by the team before their use, and whether data charting was done independently or in duplicate) and any processes for obtaining and confirming data from investigators. | 8, Tables S5 and S6 |
| Data items | 11 | List and define all variables for which data were sought and any assumptions and simplifications made. | 8, Tables S5 and S6 |
| Critical appraisal of individual sources of evidence§ | 12 | If done, provide a rationale for conducting a critical appraisal of included sources of evidence; describe the methods used and how this information was used in any data synthesis (if appropriate). | Not applicable |
| Synthesis of results | 13 | Describe the methods of handling and summarizing the data that were charted. | 8 |
| **RESULTS** | | | |
| Selection of sources of evidence | 14 | Give numbers of sources of evidence screened, assessed for eligibility, and included in the review, with reasons for exclusions at each stage, ideally using a flow diagram. | 10, Figure 1 |
| Characteristics of sources of evidence | 15 | For each source of evidence, present characteristics for which data were charted and provide the citations. | Tables 1a and 1b |
| Critical appraisal within sources of evidence | 16 | If done, present data on critical appraisal of included sources of evidence (see item 12). | Not applicable |
| Results of individual sources of evidence | 17 | For each included source of evidence, present the relevant data that were charted that relate to the review questions and objectives. | 11-16, Table 2 |
| Synthesis of results | 18 | Summarize and/or present the charting results as they relate to the review questions and objectives. | 11-16 |
| **DISCUSSION** | | | |
| Summary of evidence | 19 | Summarize the main results (including an overview of concepts, themes, and types of evidence available), link to the review questions and objectives, and consider the relevance to key groups. | 17 |
| Limitations | 20 | Discuss the limitations of the scoping review process. | 17-18 |
| Conclusions | 21 | Provide a general interpretation of the results with respect to the review questions and objectives, as well as potential implications and/or next steps. | 19-23 |
| **FUNDING** | | | |
| Funding | 22 | Describe sources of funding for the included sources of evidence, as well as sources of funding for the scoping review. Describe the role of the funders of the scoping review. | 24 |

Tricco AC, Lillie E, Zarin W, O'Brien KK, Colquhoun H, Levac D, et al. PRISMA Extension for Scoping Reviews (PRISMA-ScR): Checklist and Explanation. Ann Intern Med. 2018; 169(7):467–473

### **Table S8**: Additional results

#### **Table S8.1**: Examples of training content for health workers by RMC domains (source: Shakibazadeh et al. 2018) (n= 27 citations, 22 studies)

| **Domain** | **Number of studies** | **Focus of training content** | **Examples** |
| --- | --- | --- | --- |
| RMC domain #1- Being free from harm & mistreatment | 3 | Physical and verbal abuse | - Golden rule of NO shouting was instituted (**Oosthuizen et al., 2019**)  - A simulation training focused on a 'difficult' patient who refused to open her legs for exams and insisted on squatting to deliver. Providers were trained on how to handle this to prevent physical and verbal abuse. The PRONTO training kit was used, a hybrid birth simulator which a patient actress wears (complete with anatomical landmarks for delivery). Simulation scripts included prompts for certain behaviours from the patient actress. (**Afulani et. al, 2019**) |
| RMC domain #2- Maintaining privacy and confidentiality | 6 | Privacy during procedures, exams and delivery; maintaining confidentiality | - The admissions area was moved to a private room. A prior client service charter had been developed by community and facility stakeholders, and a facility-based quality improvement process was implemented to redefine practices and norms for RMC (**Kujawski et al., 2017**)  - Curtains and screens were procured (and existing ones repaired) to make sure that all beds had a functioning partition to provide privacy. This was done as part of action plan from the training (**Ratcliffe et al., 2016**). |
| RMC domain #3- Preserving women's dignity | 3 | Promoting women’s dignity and rights as a human being, showing respect | - Module 1 focused on respect and dignity in childbirth and covered rights of childbearing women among other things (**Dzomeku et al., 2021**)  - Main learning outcome for the training was to demonstrate link between human rights and health care, with emphasis on maternal health. Training showed how a human rights based approach may be introduced to improve experiences of women, their families and care providers (**Geddes et al., 2017**) |
| RMC domain #4- Prospective info provision & seeking informed consent | 5 | Providing information about procedures, plans, rights and seeking informed consent | - A series of interventions were done with respect to informed consent to address inadequate risk discussion and lack of women’s involvement in decision-making: use of a standardised checklist (with 5 components of the informed consent process) which was integrated into the health facility's preoperative form; wall posters placed at eye level in every labour room to serve as additional reminder to maternity staff to initiate informed consent discussion; and communication training, including role-plays for elective and unplanned C-section (**Zethof et al., 2020**) |
| RMC domain #5- Ensuring continuous access to family/ community support | 5 | Allowing birth companions, ensuring access to familial support | - Intervention facilities received workshop training on promoting childbirth companions including benefits, deciding on choices of birth companions, recruiting volunteer childbirth companions (and how to praise/comfort/support the women), and identifying and addressing obstacles (**Brown et al., 2007**)  - A wide-ranging quality improvement training was done, and a selected number of RMC ideas were selected for implemented, which included informing pregnant women about the option of having a birth companion during labour/delivery (**Mengistu et al., 2021**) |
| **Domain** | **Number of studies (ref. #)** | **Focus of training content** | **Examples** |
| RMC domain #6- Enhancing quality of physical environ./resources | 3 | Ensuring birth environment is clean/comfortable and resources such as medicines are available | - Modules on RMC were given and then a list of possible interventions was drawn and implemented, including: waiting room for pregnant and labouring mothers established (with mattresses, TV, private toilet); maternal reception room integrated with inpatient department for women who came to deliver (with food services provided); toilet having accessible door and WASH products; essential drugs, equipment and supplies provided (**Mihret et al., 2020**)  - Supply stock outs were posted to ensure transparency and trust-building. This was done so that patients and their relatives would know that providers were telling the truth (women used to think they were lying to make money for themselves, since maternity care was supposed to be free; this used to be a trigger for negative interactions). The hospital pharmacist would compile a weekly list of available and out-of-stock drugs/supplies and this was posted in the maternity ward and other key department (**Ramsey et al., 2016, Kujawski et al., 2017**) |
| RMC domain #7- Providing equitable maternity care | 0 | Providing non-discriminatory care irrespective of social class, ethnicity, age, religion or other factors | Included studies did not specifically focus on this domain but may have included it in wider training |
| RMC domain #8- Engaging with effective communication | 7 | Effective communication- both verbal and non-verbal, listening, discussing | - Focused on communicating appropriate info to key stakeholders. Focused on compassionate care, and among other things, training participants were taught how to compassionately and skilfully intervene when they witness colleagues being disrespectful to patients or other staff. They were also given and encouraged to wear 'Compassionate Care Champions' buttons as a visible way of communicating their commitment to compassionate care to patients/colleagues (**Pfeiffer et al., 2019**)  - As part of action plan developed from training, exit surveys were developed to receive more regular feedback from patients, with results shared quarterly in departmental meetings. Weekly discussion of issues relating to communication (and other topics) done during department meetings. Open Birth Days done, which included birth preparedness sessions and tour of hospital to show women wards they might encounter during childbirth (**Ratcliffe et al., 2016**)  - A training manual covering a range of communication topics was used. An observation checklist was used to assess midwife-client interaction (**Akin-Otiko and Bhengu, 2013**) |
| RMC domain #9- Respecting women's choices strengthening their capabilities to give birth | 3 | Empowering/encouraging women to make decisions, allowing mobility and freedom to choose preferred birth positions | - One module focused on effective, alternative birthing positions and covered: main cost-effective positions in childbirth (e.g. birthing stool, kneeling, side curled position, etc); advantages of these positions; skills required to teach women about these positions; and practical demonstration on using these positions during childbirth (**Dzomeku et al., 2021**)  - Training highlighted respecting women's autonomy and supporting them whichever way (e.g., encouraging birth companions). Simulation activity also trained on handling women's desires for alternative birthing positions (**Afulani et al., 2019**)  - Cultural birthing positions were allowed, and food and drinks were offered (**Oosthuizen et al., 2019**) |
| RMC domain #10- Availability of competent and motivated human resources | 6 | Ensuring staff are competent and skilled, meeting staff needs, managers providing supportive supervision, encouraging team-based care | - Counselling for providers (‘Caring for Carers’) was done as part of interventions (**Abuya et al., 2015, Warren et al., 2017**)  - Written guidelines and protocols on responsibilities of HCPs on RMC was prepared. Also regular M&E was established through mentoring, supportive supervision, and recognising good-performing employees (**Mihret et al., 2020**)  - As requested by midwives, an interactive session was held with government officials on challenges of rural midwifery. This was an opportunity for midwives to interact with their employers and express their concerns. Key issues relating to welfare of midwives and conditions of health facilities were discussed (**Akin-Otiko and Bhengu, 2013)**  - A range of actions including: maternity ward staff encouraged and reminded each other to provide respectful care; tea was provided for all shifts in the maternity; counselling (and also transfers) was done to staff who continued to show disrespectful behaviours; best practices were shared with other wards and the regional hospital; and periodic observation of maternity wards (**Kujawski et al., 2017, Ramsey et al., 2016**)  - A range of actions implemented: A new reporting structure was put in place to expedite payment of overtime allowances, with delays reduced by up to 6 months to 6 weeks; 2 staff recognition events were held to improve staff motivation, with awardees given certificates and small gifts; system put in place to make sure bread and tea were always available in break room for staff; shifts were initially increased to increase number of staff but this was reverted from 12-hour to 8-hour shifts due to overwork and complaints (**Ratcliffe et al., 2016**). |
| RMC domain #11- Provision of efficient and effective care | 3 | Avoiding procedures/exams that are unnecessary, providing timely care, managing pain | - The training- a human resources for health mentorship program- focused on reducing episiotomy rates (**Ndayambaje et al., 2017**)  - A multifaceted implementation done: staff were trained on prompt patient attention to reduce delays, among other things; A computerised automated appointment system was established to reduce wait times and enable prompt consultation. Women were able to pre-schedule visits and receive reminders (**Okonofua et al., 2020**) |
| RMC domain #12- Continuity of care | 1 | Continuity of care throughout the maternal phase, continual presence of staff during labour and delivery | - Referral of all high-risk births to the appropriate level of care was done as part of the intervention (**Oosthuizen et al., 2019**) |

####

#### **Table S8.2**: Breakdown of training content outside scope of the 12 RMC domain framework

| **A. RMC-focused but did not ‘fit’ into analytical framework**  **Rights**  - RMC standards and rights  - Clients’/patients’ and providers’ rights and obligations  - Rights-based approach  - Human rights (both as a broad/ international topic and also within the context of maternal health)  **Codes of conduct/ ethics**  - Professional codes of conduct  - Professional ethics/ practices  - Healthcare ethics  - Assessing current practice in relation to ethics  **Attitudes**  - Relationship development with women  - Cognitive behaviour techniques  - Interpersonal skills  - Skills-building on empathic communication  - Values clarification and attitude transformation (VCAT)  - Personal accountability  - Improving provider attitudes  - Conflict resolution  - Empathy  **Non-specific RMC topics**  - Patient-centred care/compassionate care/ woman-centred care/ respectful and non-abusive care/ respectful woman-centred approach in labour  - Compassionate, respectful and caring planning monitoring and implementation  - Disrespect & abuse  - Prevalence of mistreatment in the setting  - Quality maternity care provision  - Improving women’s experiences  - Reflections on patients’ personal situations/desires when they seek care  - Reflections on interpersonal/structural barriers that prevent RMC provision  - Support during childbirth | **B. Non-RMC focused**  **Broad maternal health care topics**  - Overview of maternal health  - Clinical case review  - Focused antenatal care (FANC)  - Continuous quality improvement  - Clinical mentorship  - BEmONC  - Bedside teaching  - Best practices  - Treatment algorithms for clinical decision-making  - Pregnancy complication management  - Maternal death reviews and surveillance  - Birth preparedness  **Healthcare management**  - Leadership  - Adaptive decision-making  - Wider components of Human Resources for Health  - Theory exposure/ re-exposure  - Counselling methods  - Collaborative in-services  - Respectful and shared decision-making with clients  - Inter-professional collaboration  - Problem-solving skills  - Sharing experiences and deconstructing phenomenon of abuse  - Health Workers for Change curriculum (used in a few studies; has 6 workshops-"Why I am a health worker?"; "How do our clients see us"; "Women's status in society"; "Unmet needs"; "Overcoming obstacles at work"; and "Solutions" |
| --- | --- |

#### **Table S8.3**: Overview of training stakeholders in included studies (n= 27 citations, 22 studies)

| **Category** | **Frequency in included studies (%)** |
| --- | --- |
| **Commissioner(s) of training^#^** |  |
| Researchers/university groups/research institutes | 4 (18.2) |
| Health facility management | 1 (4.5) |
| Government | 4 (18.2) |
| International organizations | 5 (22.7) |
| Local NGOs/groups | 4 (18.2) |
| Not reported/unclear | 12 (54.5) |
| **Make-up of commissioner(s) of training** |  |
| Single commissioner | 4 (18.2) |
| Multiple commissioners | 6 (27.3) |
| Not reported | 12 (54.5) |
| **Funder(s) of training^#^** |  |
| Researchers/universities/research institutes | 4 (18.2) |
| Health facility management | 1 (4.5) |
| Government | 2 (9.1) |
| International organizations | 16 (72.7) |
| Local NGOs/groups | 0 (0.0) |
| Not reported/unclear | 4 (18.2) |
| **Make-up of funder(s) of training** |  |
| Single funder | 8 (36.4) |
| Multiple funders | 10 (45.5) |
| Not reported/unclear | 4 (18.2) |
| **Trainer organization/personnel^#^** |  |
| Researchers/educators/ university groups/ research institutes | 12 (54.5) |
| Health workers/health facility management | 12 (54.5) |
| Government/policy makers | 6 (27.3) |
| International organizations | 7 (31.8) |
| Local NGOs/groups | 5 (22.7) |
| Community members | 2 (9.1) |
| Not reported/unclear | 1 (4.5) |

*# All that applied ticked, hence numbers may not add up to 22.*

* Many studies included multiple training roles/methods, which meant that there was a range of training organizations/personnel. A few studies set up committees or multidisciplinary teams for the training.

Additional notes on categories for commissioners, funders and trainers: ‘Researchers/university groups/research institutes’ also includes institutions such as Ifakara Health Institute, JHPIEGO, South Africa MRC; ‘Government’ also included Ministries of Health; ‘International organizations’ were mostly governmental international organizations such as USAID and DFID, but also included professional international organizations such as FIGO; ‘Local NGOs/groups’ also included groups such as local professional organizations (such as the National Nursing Association of Kenya, the Society of Gynaecologists and Obstetricians of Burkina Faso or Federation of Women Lawyers in Kenya).

Summaries have been included below for categories where there is some information in a few studies, but not uniform enough across studies to be summarised in a meaning way in a table (most of these categories were largely not reported in the studies).

- **Sex of trainer(s)**: Only one study reported (Ramsey et al., 2016). It reported that the training facilitator was a female facilitator.
- **Experience level of trainers**: Many studies did not report level of experience of the trainers; only about a third did (7 studies). For the studies who reported, trainers appeared to be very experienced. For example, they were part of professional organizations such as the country’s midwives’ association or nursing council, were senior staff, or had advanced degree. A few of the studies only said trainers ‘were experienced’ or ‘had significant experiences in such and such’.

#### **Table S8.4**: Overview of trainees in included studies (n= 27 citations, 22 studies)

| **Category** | **Frequency in included studies (%)** |
| --- | --- |
| **Total number of health workers trained** |  |
| <30 | 4 (18.2) |
| 30-59 | 4 (18.2) |
| 60-80 | 4 (18.2) |
| 81-100 | 0 (0.0) |
| >100 | 5 (22.7) |
| Not reported/unclear | 5 (22.7) |
| **Health worker roles trained^+^** |  |
| Direct care only (clinical) | 18 (81.8) |
| Operations only (non-clinical) | 0 (0.0) |
| Both direct care and operations | 4 (18.2) |
| **Cadre of health workers trained^#^** |  |
| Midwives | 15 (68.2) |
| Nurses | 8 (36.4) |
| Doctors/physicians/surgeons | 10 (45.5) |
| Other/broad clinical^ | 8 (36.4) |
| Non-clinical | 3 (13.6) |
| **Make-up of cadres in training** |  |
| Single cadre only^*^ | 8 (36.4) |
| Mixed cadres | 14 (63.6) |

*+ For health workers in leadership roles, these were categorised as “direct care” if these were clinical leadership roles (e.g. maternity-in-charges), and as “operations” if these were non-clinical (e.g. district-level admin, county health managers)*

*# All cadres that applied were ticked.*

*^ ‘Other/broad clinical’ included a range of cadres such as associate clinicians/non-physician clinicians, facility in-charges, or ‘maternity staff’*

* *Only one cadre tended to be trained in these studies (e.g. midwives only, nurses only)*

Summaries have been included below for categories where there is some information in a few studies, but not uniform enough across studies to be summarised in a meaning way in a table (most of these categories were largely not reported in the studies).

- **Age of health workers**: Four studies provided ages of health workers trained, which shows a young demographic group overall. Breakdown given below in the 4 studies:
  - Range 32-59 years
  - 73.3% of the health workers were between 22-29 years
  - 33 years on average (range 31-48 years)
  - Mean age of providers was 35.2 years
- **Sex of health workers**: Three studies reported sex of the health workers, which shows that the providers were overwhelmingly female: 89% in one study, 68.8% in another, and 52 women and 8 men in the third study.
- **Ethnicity**: No study reported on ethnicity (*one study mentioned religion of the health workers, which can sometimes serve as a proxy for ethnicity, but perhaps not relevant in this context*)
- **Level of health care experience/ number of years in service** *(categories merged here since similar)*: Six studies reported. Shows varying levels of experience across studies. Breakdown below:
  - 72% of the 35 providers who took baseline survey had never had simulation-based training before
  - 4-30 years of experience
  - 50% had served as health professionals for >5 years and 39.1% had served in their current hospital for <2 years
  - 8 years of professional practice on average
  - One year was median time respondents had spent working in their respective wards
  - Authors said the midwives were experienced
- **Nature of role**: Only one study reported on the nature of the health workers’ role: 62.5% on average reported working 3 or more weekend or night shifts per week

**References**

1. Abuya, T., C. Ndwiga, J. Ritter, et al. The effect of a multi-component intervention on disrespect and abuse during childbirth in Kenya*.* *BMC Pregnancy Childbirth* 2015;15:224.

2. Warren, C.E., C. Ndwiga, P. Sripad, et al. Sowing the seeds of transformative practice to actualize women's rights to respectful maternity care: reflections from Kenya using the consolidated framework for implementation research*.* *BMC Womens Health* 2017;17(1):69.

3. Afulani, P.A., R.A. Aborigo, D. Walker, et al. Can an integrated obstetric emergency simulation training improve respectful maternity care? Results from a pilot study in Ghana*.* *Birth* 2019;46(3):523-32.

4. Akin-Otiko, B.O. and B.R. Bhengu. Building capacity of midwives for result-oriented client education and friendly service*.* *West African Journal of Nursing* 2013;24(1):28-41.

5. Asefa, A., A. Morgan, M.A. Bohren, et al. Lessons learned through respectful maternity care training and its implementation in Ethiopia: an interventional mixed methods study*.* *Reprod Health* 2020;17(1):103.

6. Asefa, A., A. Morgan, S. Gebremedhin, et al. Mitigating the mistreatment of childbearing women: evaluation of respectful maternity care intervention in Ethiopian hospitals*.* *BMJ Open* 2020;10(9):e038871.

7. Brown, H., G.J. Hofmeyr, V.C. Nikodem, et al. Promoting childbirth companions in South Africa: a randomised pilot study*.* *BMC Med* 2007;5:7.

8. Dzomeku, V.M., A.B. Boamah Mensah, E.K. Nakua, et al. Midwives' experiences of implementing respectful maternity care knowledge in daily maternity care practices after participating in a four-day RMC training*.* *BMC Nurs* 2021;20(1):39.

9. Dzomeku, V.M. Development of a patient-centred in-service training programme for midwives to increase client satisfaction with child-birth care in Kumasi, Ghana,. 2016. University of the Western Cape: Western Cape.

10. Geddes, J., T. Humphrey, and R.M.M. Wallace. Respectful midwifery care in Malawi: A human rights-based approach. *African Journal of Midwifery and Women's Health* 2017;11(4):196-98.

11. Honikman, S., S. Field, and S. Cooper. The Secret History method and the development of an ethos of care: Preparing the maternity environment for integrating mental health care in South Africa*.* *Transcult Psychiatry* 2020;57(1):173-82.

12. Mengistu, B., H. Alemu, M. Kassa, et al. An innovative intervention to improve respectful maternity care in three Districts in Ethiopia*.* *BMC Pregnancy Childbirth* 2021;21(1):541.

13. Mihret, H., A. Atnafu, T. Gebremedhin, et al. Reducing Disrespect and Abuse of Women During Antenatal Care and Delivery Services at Injibara General Hospital, Northwest Ethiopia: A Pre-Post Interventional Study*.* *Int J Womens Health* 2020;12:835-47.

14. Ndayambaje, A., R. Anderson, C.M. Yoder, et al. Human Resources for Health (HRH) Midwives Mentoring Program and episiotomy rates at Muhima Hospital, Rwanda: A retrospective cross-sectional study *Rwanda Medical Journal* 2017;74(2):1-11.

15. Okonofua, F.E., L.F.C. Ntoimo, B.F. Ekezue, et al. Outcomes of a multifaceted intervention to improve maternal satisfaction with care in secondary hospitals in Nigeria*.* *Glob Health Action* 2020;13(1):1856470.

16. Oosthuizen, S.J., A.M. Bergh, J. Grimbeek, et al. CLEVER maternity care: A before-and-after study of women's experience of childbirth in Tshwane, South Africa*.* *Afr J Prim Health Care Fam Med* 2020;12(1):e1-e8.

17. Oosthuizen, S.J., A.M. Bergh, J. Grimbeek, et al. Midwife-led obstetric units working 'CLEVER': Improving perinatal outcome indicators in a South African health district*.* *S Afr Med J* 2019;109(2):95-101.

18. Ouedraogo, A., S. Kiemtore, H. Zamane, et al. Respectful maternity care in three health facilities in Burkina Faso: the experience of the Society of Gynaecologists and Obstetricians of Burkina Faso*.* *Int J Gynaecol Obstet* 2014;127 Suppl 1:S40-2.

19. Pfeiffer, E., M. Owen, C. Pettitt-Schieber, et al. Building health system capacity to improve maternal and newborn care: a pilot leadership program for frontline staff at a tertiary hospital in Ghana*.* *BMC Med Educ* 2019;19(1):52.

20. Kujawski, S.A., L.P. Freedman, K. Ramsey, et al. Community and health system intervention to reduce disrespect and abuse during childbirth in Tanga Region, Tanzania: A comparative before-and-after study*.* *PLoS Med* 2017;14(7):e1002341.

21. Ramsey, K., W. Moyo, A. Larsen, et al. Staha Project: Building understanding of how to promote respectful and attentive care in Tanzania (Implementation research report). 2016. Ifakara Health Institute, USAID, Averting Maternal Death and Disability (Mailman School of Public Health, Columbia University): Ifakara, Washington DC, New York

22. Ratcliffe, H.L., D. Sando, G.W. Lyatuu, et al. Mitigating disrespect and abuse during childbirth in Tanzania: an exploratory study of the effects of two facility-based interventions in a large public hospital*.* *Reprod. Health* 2016;13:13.

23. Shimoda, K. and M. Lida. Respectful maternity care (RMC) seminar in Tanzania*.* *St. Luke’s International University, Tokyo Asia Africa Midwifery Research Center Newsletter* 2018;24:1-3.

24. Umbeli, T., I. Murwan, A. Kunna, et al. Impact of health care Provider’s training on patients’ communication during labor at Omdurman maternity hospital, Sudan 2011*.* *Sudan J Med Sci* 2014;9(4):211-16.

25. Webber, G., B. Chirangi, and N. Magatti. Promoting respectful maternity care in rural Tanzania: nurses' experiences of the "Health Workers for Change" program*.* *BMC Health Serv. Res.* 2018;18:6.

26. Wilson-Mitchell, K., J. Robinson, and M. Sharpe. Teaching respectful maternity care using an intellectual partnership model in Tanzania*.* *Midwifery* 2018;60:27-29.

27. Zethof, S., W. Bakker, F. Nansongole, et al. Pre-post implementation survey of a multicomponent intervention to improve informed consent for caesarean section in Southern Malawi*.* *BMJ Open* 2020;10(1):e030665.
